# Proteomic and structural differences in lumpfish skin among the dorsal, caudal and ventral regions

**DOI:** 10.1038/s41598-019-43396-z

**Published:** 2019-05-06

**Authors:** Deepti M. Patel, Katarina Bhide, Mangesh Bhide, Martin H. Iversen, Monica F. Brinchmann

**Affiliations:** 1 Faculty of Biosciences and Aquaculture, Nord University, 8049 Bodø, Norway; 2 Laboratory of Biomedical Microbiology and Immunology, 73, 04181, University of Veterinary Medicine and Pharmacy, Košice, Slovakia

**Keywords:** Proteomics, Innate immunity, Mucosal immunology, Proteomics

## Abstract

Fish skin is a vital organ that serves a multitude of functions including mechanical protection, homeostasis, osmoregulation and protection against diseases. The expression of skin proteins changes under different physiological conditions. However, little is known about differences in protein expression among various body sites in naïve fish. The objectives of this work is to study potential differences in protein and gene expression among dorsal, caudal and ventral regions of lumpfish skin employing 2D gel based proteomics and real-time PCR and to assess structural differences between these regions by using Alcian blue and Periodic acid Schiff stained skin sections. The proteins collagen alfa-1, collagen alfa-2, heat shock cognate 71 kDa, histone H4, parvalbumin, natterin-2, 40S ribosomal protein S12, topoisomerase A and topoisomerase B were differentially expressed among the three regions. mRNA expression of *apoa1*, *hspa8* and *hist1h2b* showed significant differences between regions. Skin photomicrographs showed differences in epidermal thickness and goblet cell counts. The ventral region showed relatively high protein expression, goblet cell count and epidermal thickness compared to dorsal and caudal regions. Overall, this study provides an important benchmark for comparative analysis of skin proteins and structure between different parts of the lumpfish body.

## Introduction

Skin is the largest and outermost organ in the fish body. It is the first protective barrier between its internal organs and the external aquatic environment^1^. Fish skin is a multifunctional tissue that is involved in communication, excretion, maintains the ionic balance by osmoregulation, provides mechanical support, and serves as a barrier against physical abrasion, environmental toxins and physiological stress responses^2,3^. Knowledge on fish skin is not only important for fish health and welfare, but it is also important in mucosal research on higher vertebrates as it shares many features with mammalian gut^4^.

Lumpfish (*Cyclopterus lumpus*), is a scorpaeniform fish distributed throughout the Norwegian Sea. This fish is widely used as a biological tool for sea lice removal in Atlantic salmon farms^5^. Although lumpfish is very popular as a powerful weapon against sea lice, this species is poorly studied in terms of its biology and molecular factors. A better understanding on the biology of this species is needed to establish effective welfare practices in intensive farming. Lumpfish has rough scaleless skin and little mucus compared with other species like Atlantic cod (*Gadus morhua*) and Atlantic salmon (*Salmo salar*) (our own unpublished observation). The skin integrity and optimal function are even more important for the health and welfare perspective in fishes than in mammals, as the fish skin is a mucosal surface constantly submerged in water and exposed to biotic and abiotic factors. The teleost skin is mainly composed of two layers, the outer epidermis and inner dermis, and an outer mucus layer that acts as primary line of defence against pathogens^4^.

The mucus is produced by goblet cells present in the epidermis of the skin. It is known that goblet cell number can vary among different regions of the fish body^6^, it can also vary depending on the physiological conditions of fish^1,7^. In Atlantic salmon, mucous cell density are higher in the dorsolateral skin than on the head^6^. In brown trout (*Salmo trutta*) and char (*Salvelinus alpinus*) there are more mucous cells in the anterior part of the fishes^8^. Different skin areas can vary in thickness and gilthead sea bream (*Sparus aurata*) has thicker ventral epidermis than dorsal epidermis^9^. Differences in gene expression among various body parts have also been observed^10^. In Atlantic cod (*Gadus morhua*) differential expression of immune and stress genes were observed with a higher gene expression on the ventral side compared to the dorsal side^10^. Rainbow trout (*Oncorhynchus mykiss*) had more immune relevant gene expression in the skin close to the gills compared to the skin in anterior regions^11^. In gilthead seabream there were however no differences in the expression of the immune genes studies between the vertical and dorsal areas^9^. In Atlantic salmon, differences in immune gene expression were observed in scaled compared to scaleless areas^12^. In goldfish (*Carassius auratus*) a study was focused on the pigment pattern in dorso-ventral region of skin by analysing the differential expression pattern of agouti signalling protein. The agouti signalling protein could inhibit melanisation and was found highly expressed in the ventral compared to the dorsal area in gold fish skin^13^.

To assess the protein expression, omics technologies provide a suitable platform to explore the skin associated defence factors of non-model species like lumpfish that have very limited gene and protein information available in various databases^14,15^. We have earlier identified proteins in lumpfish skin mucus^16^, among these some of the proteins have been found in other teleost species to have a role in immune defence^1,15^. Skin/skin mucus proteomic analyses have also been performed in other teleost species under normal physiological conditions such as Atlantic cod^17^, European sea bass (*Dicentrarchus labrax*)^18^, gilthead sea bream^19^, and marine catfish (*Cathorops spixii*)^20^. Thus, the present study aims to investigate the differences in histological skin sections using Alcian blue and Periodic acid Schiff staining, protein expression using two dimensional gel electrophoresis coupled with tandem mass spectrometry, and the gene expression of selected genes using qPCR in lumpfish skin among different body regions. To our knowledge this is the first study approaching a differential protein expression among various body sites in fish.

## Results

### Mass spectrometry analysis of lumpfish skin samples

**Figure 1:**
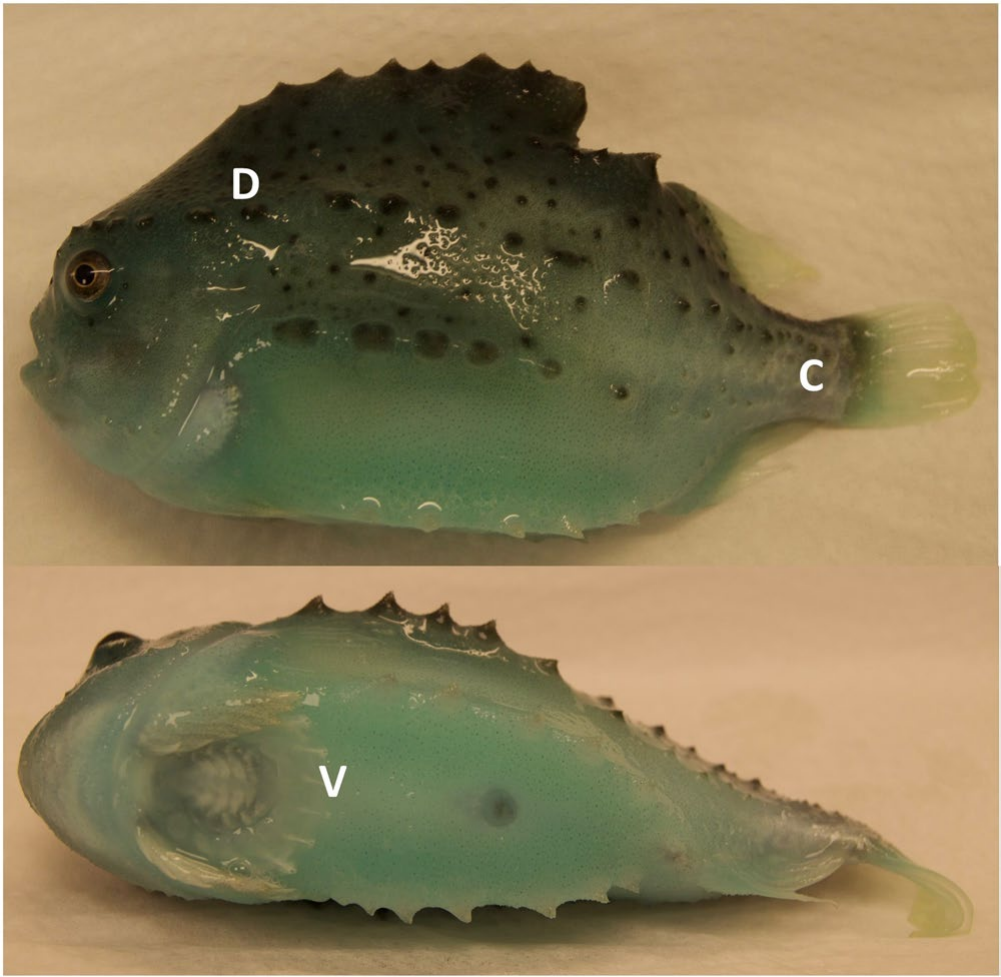
Lumpfish image indicating different regions of skin sampled in this study. Skin from three regions was sampled for proteomics, mRNA expression and histology in this study. D; dorsal region above the lateral line near operculum, C; caudal region below the lateral line near caudal peduncle, V; ventral region near the adhesive disc.

**Figure 2:**
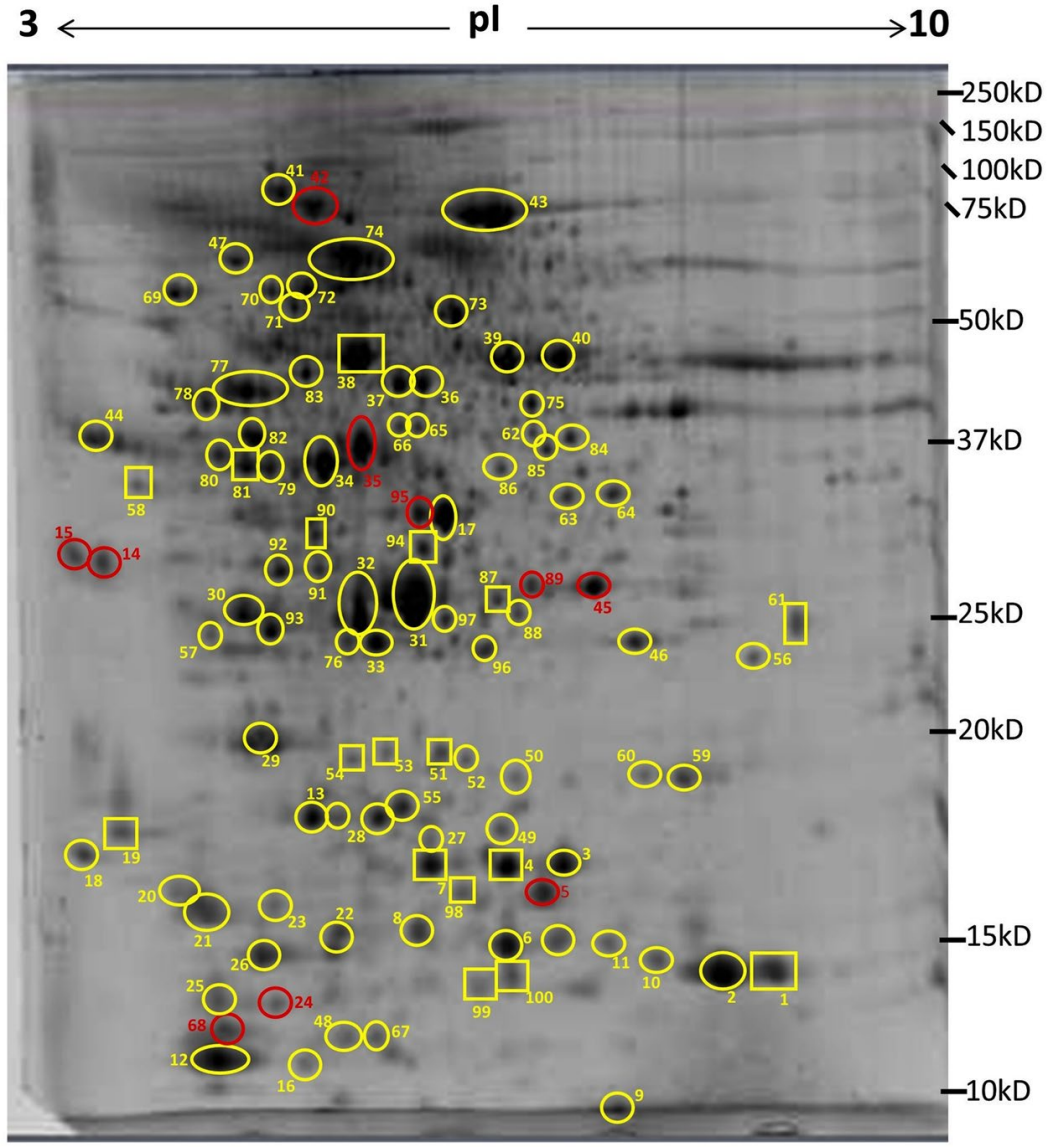
A 12.5% acrylamide gel image showing spots that were excised and sequenced. Spots circled in yellow are identified but not differentially expressed, spots circled in red are identified and differentially expressed, spots in yellow rectangles are not identified neither differentially expressed, spots in red rectangles are not identified but differentially expressed. Protein identities are found in Supplementary Table S1.

**Figure 3:**
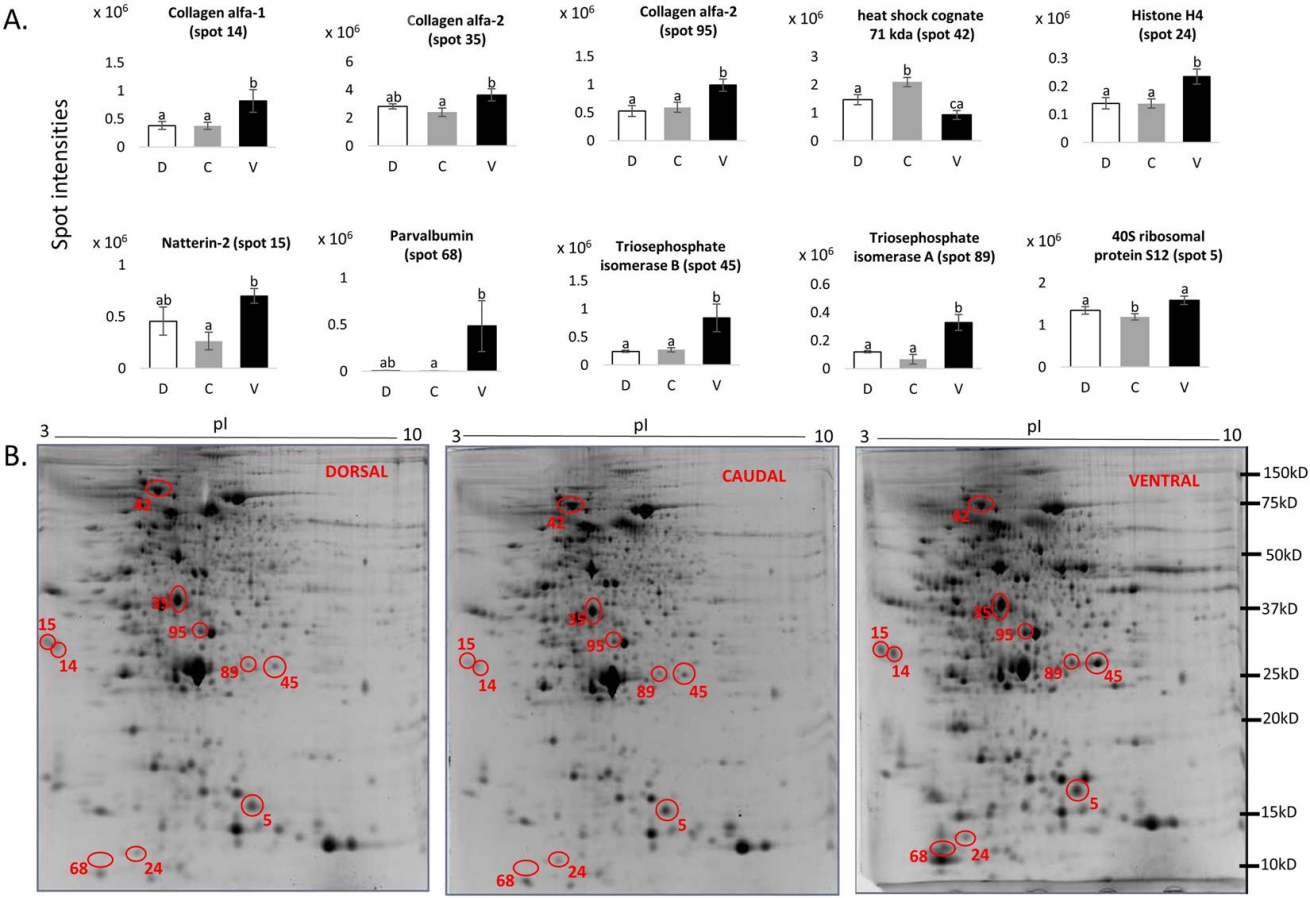
Differentially expressed skin proteins shown as spot intensities in bar graphs and spots in 12.5% acrylamide gels (**A**) Spot intensities (X-axis) of differentially expressed proteins among dorsal (D), caudal (C) and ventral (V) region of lumpfish skin based on PDQuest analysis. One way ANOVA and Tukey HSD post hoc analysis were used for normally distributed data whereas Kruskal-Wallis test and Dunn’s test were used for non parametric data. Error bar shows the error of mean. Bars with different letters are significantly different, p < 0.05, *n* = 6. (**B)** Gel images from dorsal, caudal and ventral regions showing the differentially expressed proteins. Spots are encircled in red and assigned by specific spot numbers.

In this study we have identified proteins in lumpfish skin by using 2D gel electrophoresis coupled with liquid chromatography and tandem mass spectrometry. This method along with homology search is a very useful tool for proteome analyses of species like lumpfish with very little molecular data available in public databases. A total of 18 gels, 3 gels per each of 6 fish were run. Per fish, one gel was run from each region. The three regions were i. the dorsal region (D) above the lateral line close to operculum, ii. the caudal region (C) below the lateral line close to caudal peduncle, iii. the ventral region (V) near the adhesive disc. Electronic images of the gels were captured and used for PDQuest analysis (BioRad) (Fig. 1). Seventeen differentially expressed spots among the three different skin regions were excised, subjected to LC-MS/MS and identified using MASCOT. Of 17 spots, 10 spots were matched to specific protein hits, the rest did not show any match in the homology search (Figs 2 and 3). The peptides from unmatched protein spots were blasted in NCBI protein blast, but it did not give unambiguous results. In addition to the differentially expressed spots we also excised 83 spots that were expressed in all three regions in high density (Fig. 2), to create a protein reference map of lumpfish skin. This provides additional molecular information of lumpfish skin, which could be used as a benchmark for further comparison studies under various physiological conditions. Details of the identified proteins are listed in Supplementary Table S1. Proteins that had significant differential expression among the three different skin regions are shown in Fig. 3. All gel images are provided as Supplementary Fig. 1.

### Gene ontology and protein interaction

Gene ontology terms of biological process for the identified proteins were retrieved manually from UniProt. As most of the proteins are not well annotated in teleost species the gene ontology terms were retrieved from its human counterparts (Table S1).

**Figure 4:**
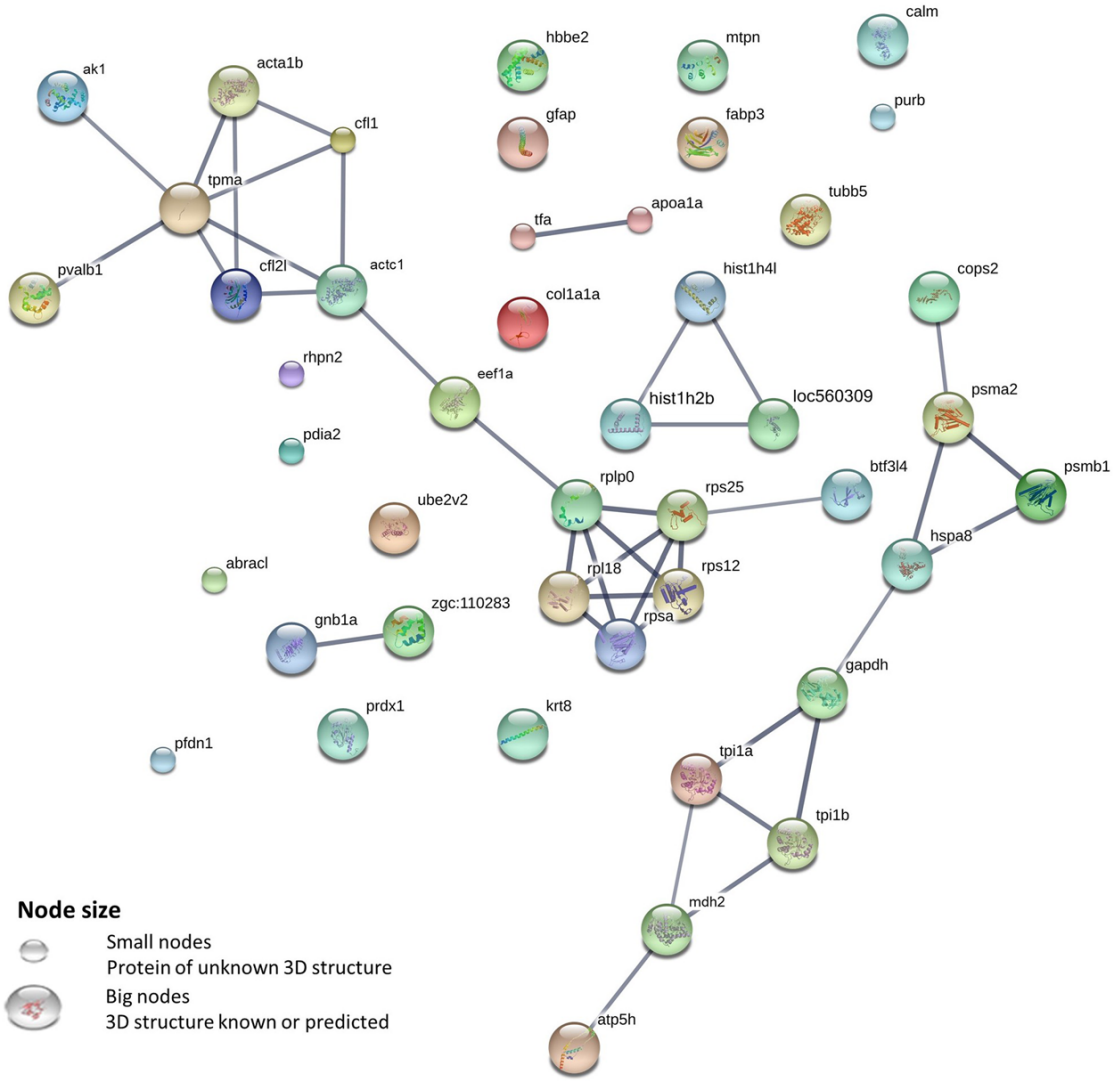
Protein interaction map of identified lumpfish skin proteins. A possible protein-protein interaction map with high edge confidence was generated by string v.10. Thicker edges (line joining the nodes) represent a confidence of 0.900/1. Slightly thinner edges represent a confidence of 0.700/1. Edges represent protein-protein association where association does not necessarily mean physical binding of the proteins, there could be involvement of several proteins to a shared function. Protein interaction network is created using zebrafish orthologues of the proteins identified in lumpfish skin. Full protein name for the abbreviation are provided as Supplementary Table S2. Role of these proteins are mentioned in results and discussion sections.

A possible protein-protein interaction map was created employing zebrafish orthologues with high edge confidence level (<0.700) using string v10.0 (Fig. 4). The protein interaction network created 45 nodes and 39 edges with an average node degree of 1.73. The network highlights the interaction of ribosomal proteins (rpsa, rplp0, rps12, rpl18, rps25), histone proteins (hist1h41, cr762436.3, Loc560309), cytoskeletal proteins (actc1, acta1b, tpma, cfl2l, cfl1), enzymes (ak1, mdh2, gapdh, tpi1a, tpi1b, atp5h), nucleotide binding proteins (gnb1a, zgc:110283), proteasome subunits (psmb1, psma2), parvalbumin, apolipoprotein A1 and transferrin. Full protein names of the abbreviations used in the protein interaction network are provided as Supplementary Table S2. All abbreviations of the protein in the interaction map are assigned by string.

### mRNA expression levels of selected genes in lumpfish skin

Several proteins were identified in the skin proteome of lumpfish. After protein identification, quantitative real time PCR has been widely used as a complementary technique to analyse whether local syntheses of the proteins are possible or not. The target genes were selected for analysis based on their suggested functions in teleost, as knowledge about the skin in lumpfish is scarce. Due to the fact that some previous studies in other species^10–12^ have found differences in the expression of some of these gene in different areas of fish skin, whilst others found no differences^9^, we aimed to analyse the mRNA expression level of a few genes suggested to be immune and/or stress relevant in other fish species.

Thus, among the differentially expressed proteins (Fig. 3) we picked heat shock cognate 71 kDa (*hspa8*) and natterin-2. In addition, we also selected apolipoprotein A1 (*apoa1*), calmodulin (*calm*) and histone H2B (*hist1h2b*) based on their recurring identification in skin and mucus studies according to the available literature^21–23^. Three reference genes glyceraldehyde 3-phosphate dehydrogenase (*gapdh*), beta actin (*β-actin*), elongation factor 1 alfa (*ef1-alfa*) were selected and verified for this study. Due to unavailability of lumpfish gene sequences in public databases we designed the primers using sequences from other teleost species focusing on conserved regions. The primers for the reference genes did not have degeneracy and were used for the real time assay (Table 1). The target genes showed degeneracy, thus degenerate PCR was opted for the target genes. The degenerate primers used in this study are found in Table 1. Four genes (*hspa8*, *apoa1*, *calm*, *hist1h2b*) were amplified successfully, but natterin-2 did not show any amplification. Therefore, we proceeded with 4 target genes *hspa8*, *apoa1*, *calm* and *hist1h2b* for the real time quantitative PCR. In order to design the real time PCR primers we had to sequence the PCR products obtained from the degenerate PCR of *hspa8*, *apoa1*, *calm* and *hist1h2b*.

**Table 1:** Oligonucleotide sequences used in the study.

Gene name	Primer sequence	Amplicon size	Primer efficiency (%)	Purpose of use
*apoA1*	F: TACMTRRCTCRGGTGAARGASA	528	—	Degenerate PCR
	R: CTTGTAYTCYKSARCRTAGGG			
	F: ACATGCACACCAAGCTCAG	107	98.678	qPCR
	R: AATGATTGAGGAGCGGAAG			
*calm*	F: CAGATTGCHGARTTYAARGARGC	392	—	Degenerate PCR
	R: GTTGACCTGDCCRTCWCCRT			
	F: ACGGACAGTGAGGAGGAGA	110	87.770	qPCR
	R: TTCTCCCCGAGGTTAGTCA			
*hspa8*	F: GGCACTACCTACTCCTGTGTAG	706	—	Degenerate PCR
	R: TTTGAACTCRGAGATGAAGTGG			
	F: TCTCATTGGACGTCGGTTT	119	101.439	qPCR
	R: TGGTCTCGCCCTTGTACTC			
*hist1h2b*	F: ACCAGGAAGGAGAGCTATGCYATC	268	—	Degenerate PCR
	R: CTTGGTGACGGCCTTKGTDC			
	F: ATCTTTGAGCGCATCGCCG	144	104.115	qPCR
	R: TGTTCCCTCAGACACCGCG			
*gapdh*	F: GCCATCAAYGACCCMTTCAT	380	—	Degenerate PCR
	R: GCAGTTRGTVGTGCAGGADG			
	F: GGGGCAAGCTCATCGTCG	149	104.03	qPCR
	R: CCTGGATGTGAGAGGAGGCC			
*β-actin*	F: GACTACCTCATGAAGATCCTGA	188		qPCR
	R: GGTGATGACCTGTCCGTC			
*ef1-alfa*	F: AAGTTCGAGAAGGAAGCCGC	98	100.584	qPCR
	R: ATGGTGATACCACGCTCACG			
*pentraxin*	F: ATCATCCTGTTYGCSTAYCGAC	Did not show amplification		
	R: GTCCCACAWKTKCASVTYWGHMA			
natterin-2	F: GCAGAGACCTGGACCAAGACGT	Did not show amplification		
	R:GCTTCTCCCTTCTATGGTACCC			

All genes were amplified for melt curve analysis. A single peak was obtained in most of the genes whereas in a few, a small peak of primer dimer was detected that was also observed in the negative control. Negative controls did not show any amplification. PCR products obtained by using real time primers for both reference and target genes were sequenced for verification of identity.

All three reference genes showed a Cq value ranging from 18–24. BestKeeper ^©^ software (http://www.gene-quantification.com/bestkeeper.html) was used to analyse the expression stability of candidate reference genes. As per the analysis the coefficient of correlation (*r*) of *gapdh*, *β-actin*, *ef1-alfa* genes are 0.930, 0.882, 0.865 respectively.

The detailed analysis obtained from BestKeeper are available as Supplementay Table S3. As all three genes did not show much variation in their expression pattern, the geometric mean of Cq obtained from *gapdh*, *β-actin* and *ef1-alfa* was used to analyse the relative expression level of target genes.

**Figure 5:**
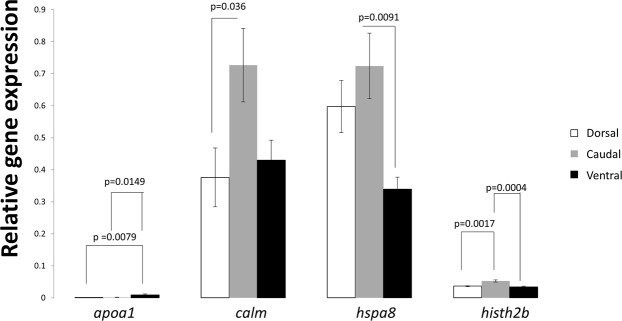
mRNA expression level of immune and/or stress related genes in teleosts. mRNA expression level of selected genes between the three different regions (D, C, V). The expression is relative to the geometric mean of three reference genes *ef1-alfa* (elongation factor alfa1), *β-actin* (beta actin) and *gapdh* (glyceraldehyde 3-phosphate dehydrogenase). Target genes in X-axis are *apoa1* (apolipoprotein A1), *calm* (calmodulin), *hspa8* (heat shock cognate 71 kDa), *hist1h2b* (histone h2b). Analysis was performed by employing one-way ANOVA (analysis of variance) followed by Tukey HSD (honest significant difference) post hoc analysis for comparison of expression between the three regions (*n* = 6), p values are denoted in the figure wherever the difference is significant. Error bars represent mean ± SE.

The expression patterns of selected target genes (*apoa1*, *hspa8*, *calm*, *hist1h2b*) for real time quantitative PCR are shown in Fig. 5. Significant differential expression of *apoa1* mRNA was detected in the ventral region compared to the dorsal and caudal regions. *calm* mRNA expression in the caudal region was significantly different compared to the dorsal region. There were no other significant differences in *calm* expression between regions. *hspa8* mRNA expression in the ventral region was significantly different from the caudal region. It did not show any significant difference between dorsal and caudal/ventral region. *hist1h2b* mRNA expression in caudal region was significantly different from the ventral/dorsal region where as there was no significant difference between dorsal and ventral region.

### Histological findings

**Figure 6:**
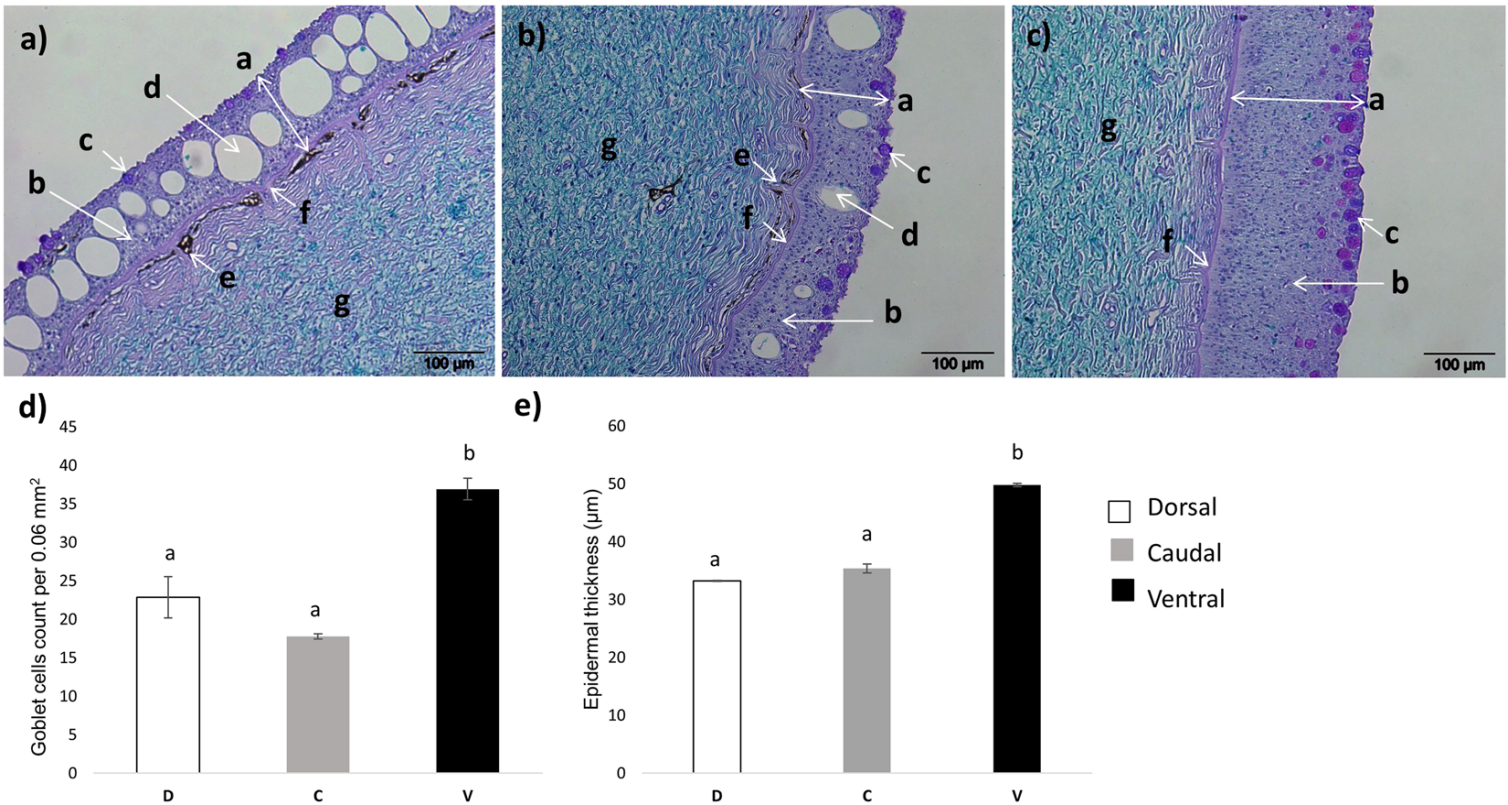
Lumpfish skin sections showing structural differences among dorsal, caudal and ventral regions of skin. Representative photomicrographs (20x magnification) of dorsal **(a)**, caudal **(b)** and ventral **(c)** regions of lumpfish skin sections stained with Alcian blue and Periodic acid Schiff. The letters represent different cells and layers in skin. (**a**) epithelial layer, (**b**) epidermal squamous epithelial cells, (**c**) goblet cells, (**d**) saccular cells (**e**) dermal pigment deposition (**f**) basal layer (**g**) dermis. Differences in goblet cell counts/0.06 mm^2^ **(d)** and epidermal thickness (µm) **(e)** between the three skin regions D (dorsal), C (caudal), V (ventral) are shown. Error bars represent mean ± SE, n = 6, p < 0.05.

The histological analysis of lumpfish skin showed that epidermal thickness (Fig. 6e) and goblet cell count (Fig. 6d) were significantly higher in the ventral region compared to the dorsal and caudal regions. Goblet cells were predominantly found towards the outer layer of epidermis. In the dorsal and caudal region we observed many cells similar to saccular cells (Fig. 6a,b), but these cells were absent in ventral region (Fig. 6c). Melanin deposition was observed in upper layer of dermis in dorsal and caudal region (Fig. 6a,b), but not in the vertical region.

## Discussion

Skin is a vital organ in fish that serves an array of functions to maintain homeostasis. It provides mechanical protection, protects the internal organs from external aqueous environment, involved in osmoregulation, immune response and sensory activities. It contains proteins, such as structural proteins, antimicrobial proteins, lectins, immunoglobulins, complement factors, proteases, and acute phase proteins^15^. In this study, we have showed the presence of several putative proteins in the skin of lumpfish creating a proteome reference map for lumpfish skin (Supplementary Table S1) using two dimensional gel based proteomics. When genomic resources to produce antibodies become available, the identity of the proteins could be verified by western blotting. Seventeen of the protein spots were differentially expressed in the skin of lumpfish among three different regions (D, C, V). There are very few studies that have focused on differences among different regions in fish skin at the molecular level. Studies on Atlantic cod^10^, rainbow trout^11^, and Atlantic salmon^12^ reported differential expression of several immune and stress related genes in different skin areas of fish. In gilthead sea bream, there were, however, no differences in the expression of the immune genes studies between the ventral and dorsal areas^9^. Furthermore, another study showed difference in expression levels of some immune genes in skin cells of Atlantic cod isolated from dorsal and ventral regions after probiotics-pathogen interactions^24^. Expression of agouti gene have been evaluated to study the dorso-ventral pigmentation pattern in gold fish using northern blot^13^, and in flatfishes (*Scopthalmus maximus* and *Solea senegalensis*) using quantitative real time PCR^25^. However, no study has been carried out to assess the difference of protein expression among various regions in fish skin.

In the present study, a protein-protein interaction map was created using String v10.0 (www.string-db.org/) to analyse the interaction of the identified proteins in lumpfish skin. String creates the interaction map based on well-established functions of different proteins mainly in model organisms. However, there are many proteins that have more than one function. Such proteins are called moonlighting proteins, and are involved in more than one biological pathway^26^. Even if the String analysis mainly uses the main function(s) of the proteins when making the interaction map, in our discussion we have also included the multitasking activities of the proteins that were differentially expressed among the three skin regions (D, C, V).

In the present study among the differentially expressed spots, D14, D35 and D95 were identified as collagen alpha-1 and alpha-2 type proteins. Collagen has been isolated from skin of teleost species for industrial purposes as an alternative to pig and bovine collagen^27^. Collagen is an essential extracellular matrix protein in fish that provides mechanical support to maintain skin integrity, cell migration, skin development and immune responses^28,29^. An *in vitro* study on gilthead sea bream found that collagen could prime respiratory burst and regulate the phagocytic activity^29^. In this study, the alpha-1 type collagen protein, which are essential for formation of type I collagen, showed relatively higher expression in the ventral region than in the dorsal and caudal regions (Fig. 3). For the alpha-2 type collagen protein, there were a significant difference between caudal and ventral regions, but between the dorsal and ventral regions (Fig. 3). This expression could be due to the presence of the adhesive disc (modified pelvic fin) in the ventral region of lumpfish. This fish spends most of its time as a sessile organism by adhering to suitable objects rather than swimming actively. The adhesive disc in this species has very strong adhesion capacity to protect the fish against the water current. Therefore, high abundance of protein like collagen could provide mechanical strength to maintain the balance and skin integrity against strong water currents.

Spot D15 was identified as natterin, a protein with lectin like domain and a toxic domain with kinogenase activity^30,31^. In skin, the lectin like domain could recognise pathogens and the toxin domain could cause lysis of pathogenic microbes. This protein was purified with a mannose affinity column from Atlantic cod skin mucus that suggests its mannose specific domain could act as pathogen recognition receptor in the skin^32^. In the current study natterin showed a differential expression between the caudal and ventral region but did not show any significant difference between dorsal and the other two regions (Fig. 3).

Spot D24 was recognized as histone H4. Histones along with their primary nuclear functions (Fig. 4) also serve as danger associated molecular patterns when released to extracellular space. This group of protein are also involved in inflammation, cell death and immune responses^33^. Histone H4 from fresh water prawn (*Macrobrachium rosenbergii*) showed antimicrobial activity against both Gram negative and Gram-positive bacteria. Furthermore, high H4 gene expression levels were reported in gills of fresh water prawn infected with pathogens such as white spot syndrome baculovirus, *M*. *rosenbergii* noda virus, *A*. *hydrophila* and *Vibrio harveyi*^34^. Histone proteins have been identified in skin/skin mucus of several teleosts such as histone H4 in mrigal (*Cirrhinus mrigala*)^35^ and European sea bass (*D*. *labrax*)^18^, H2B like protein in channel catfish (*Ictalurus punctatus*)^21^, histone like protein in sunshine bass (*Morone saxatilis*)^36^, histone like protein and H2A in rainbow trout (*Onchorynchus mykiss*)^37,38^, histone derived antimicrobial peptides in Atlantic halibut (*Hippoglossus hippoglossus*)^39^ and coho salmon (*Onchorynchus kisutch*)^40^, and histone H2B in lumpfish^16^. In the current study, the histone H4 protein was relatively highly expressed in the ventral region compared to dorsal and caudal regions. It showed a significant difference between the ventral and the two other regions, but did not show any difference between dorsal and caudal region. In addition to histone H4, H2A (spot D21) and H2B (spot D23) were also identified in lumpfish skin in this study, but these two proteins did not show any differential expression among different skin regions. In the protein interaction map (Fig. 4), the core histone proteins H4 (hist1h41), H2A (loc560309) and H2B (cr7622436.3) showed a strong interaction of the histone proteins suggesting their primary role in nucleosome complex to form the octamer complex for DNA packaging^41^.

Spot D25 and D68 were identified as parvalbumin. This protein is a widely studied fish allergen^42^ however very little is known about its role in host defence. Pravalbumin is a calcium binding protein and hence it could inhibit bacterial growth by chelating the essential cations needed for the growth and proliferation of bacteria. This hypothesis was confirmed by a study on parvalbumin extracted from cutaneous mucus of Thamnophiine snake (*Lithobates catesbeianus*) showing antibacterial activity against *Escherchia coli*^43^. This protein is involved in intercellular calcium binding that might function in calcium ion transport during muscle relaxation^44^ in association with other cytoskeletal proteins. The protein interaction map created by String showed an interaction of parvalbumin with the structural protein complex formed by interaction of actin (acta1b, actc1), cofilin (cfl1, cfl2l) and tropomyosin (tpma) (Fig. 4). The high expression of parvalbumin in the ventral region might suggest that it has activity in muscle relaxation due to its connectivity with actin and tropomyosin and might also indicate the involvement in locomotion as the cofilin proteins have a role in cell motility^45^ (Fig. 4). Parvalbumin is also widely distributed throughout the nervous system. A study on rat visual cortex during postnatal development revealed that expression level of parvalbumin is dependent on the neuronal activity where they found a positive correlation between number of neurons and parvalbumin expression^46^. Thus, high level of parvalbumin expression in ventral region than dorsal and caudal region in skin of lumpfish (Fig. 3) could be due to the presence of adhesive disc, as skin surrounding the disc needs to be neurologically alert. Relatively high level of parvalbumin, histone H4 in ventral region of lumpfish skin (Fig. 3) could protect it from pathogenic microbes when the adhesive disc is in close contact with surfaces that may contain disease-causing agents.

Spot D42 was heat shock cognate 71 kDa protein. This protein is a member of the highly conserved heat shock protein 70 family. This is a multifunctional protein that acts as molecular chaperone, stress indicator and signalling molecule^47^. Presence of this protein/gene has been reported in skin mucus of sea lice infected Atlantic salmon analysed by microarray^48^, and skin mucus of naïve lumpfish analysed by 2D gel based proteomics^16^. Expression of this protein/gene is up-regulated during stress induced by environmental parameters^49^. Heat shock cognate 71 gene in catfish (*Clarius batrachus*) showed relatively higher level of expression in different tissues (brain, muscle, spleen, heart, liver, head kidney) under hypoxic condition than the control fish that were maintained under normal oxygen level^49^. In human heat shock cognate 71 kDa protein has found to be interacting with MHC molecules and be involved in regulation of antigen trafficking^50^. Heat shock cognate proteins serves as a link between chaperones and the proteasome^51^ for proteasome activation for degradation of misfolded proteins (Fig. 4). In this lumpfish study the interaction of heat shock cognate 71 kDa protein (indicated as hspa8 in Fig. 4) with the proteasome units (psmb1 and psma2 in Fig. 4) suggests its role in cellular protein degradation via the ubiquitin-proteasome pathway^47^. Interaction of hspa8 with gapdh (Fig. 4) also suggests its role in chaperoning activity^52^. In the present study hsc71 showed relatively high expression in caudal region compared to the other two regions, but no significant difference between the dorsal region and the ventral region (Fig. 3).

Spot D45 and D89 were identified as triosephosphate isomerase B and A respectively. These enzymes are involved in carbohydrate metabolism and isomerisation of dihydroxyacetone phosphate into glyceraldehyde-3-phosphate. Triosephosphate isomerases have been reported in skin mucus of lumpfish^16^, Atlantic cod^17^, gilthead sea bream^19,53^, and European sea bass^18^. This protein was found to be significantly up-regulated in early developmental stages of mussel (*Mytilus galloprovincialis*) due to oxidative stress induced by cadmium, where the authors concluded that up-regulation of triosephosphate isomerase could be for compensation of the energy demand induced by stress^54^. Relatively high expression of these proteins in ventral region of lumpfish skin (Fig. 3) in this study could be due to comparatively high energy demand for successful adhesion to various substrates.

In addition to differentially expressed proteins, the present study also identified abundant proteins in lumpfish skin. Several proteins that were identified in the present study, were previously reported in skin mucus of lumpfish^16^. Spot D18 was identified as calmodulin. This protein is involved in inflammatory responses, intracellular and extracellular signalling^55^ and stress responses^56^. Spots D30, D31, and D32 were identified as apolipoprotein A1. This protein is primarily involved in transportation of high-density lipoprotein particles. Antimicrobial activity of apolipoprotein A1 isolated from plasma was observed in common carp (*Cyprinus carpio*) against both Gram negative and Gram positive bacteria^22^. Increased expression of this protein was observed in skin mucus of Atlantic salmon infected by sea lice^57^ and in gill mucus of Atlantic salmon affected by amoebic gill disease^58^. Spots D43 and D44 were identified as transferrin and serotransferrin. Transferrin is well known for its role in transfer and delivery of iron to the cells. It binds to iron and makes it unavailable for bacteria and creates a bacteriostatic environment. This protein is also found to be an activator of macrophagic activity by inducing nitric oxide response in macrophages in goldfish exposed to several fish pathogens^59^. Furthermore, cleaved transferrin has found to be involved in acute inflammatory responses in goldfish injected with heat killed *Aeromonas veronii*^60^. Spot D96 was identified as natural killer enhancing factor. This protein is also known as peroxiredoxin, an antioxidant protein involved in immune responses in fish such as chaperoning, inflammatory responses upon infection, balance of reactive oxygen production to reduce oxidative stress^61^. Ribosomal proteins have been identified in skin and skin mucus of many teleost species^15^ as well as in the present lumpfish study (Table S1). In the protein interaction map the ribosomal proteins clustered together forming a ribosomal protein complex (rpsa, rplp0, rps12, rpl18, rps25) (Fig. 4). The ribosomal complex was connected to the elongation factor alfa 1 (eef1a2) implying its role in protein synthesis (Fig. 4).

In this study we have identified a total of 82 proteins (Table S2) in lumpfish skin. Seventeen proteins were found to be significantly different between the three skin regions. The identified proteins in skin have several biological functions such as immune response, response to stimulus, cytoskeletal activity, metabolic activity and others (Table S1). To further study molecular differences between skin regions mRNA expression was used. However, without genomic/transcript resources it is not easy to analyse the mRNA expression level of all the 82 genes encoding the proteins identified in lumpfish skin. Using degenerate PCR method, a few selected genes such as *apoa1*, *calm*, *hspa8* and *hist1h2b* were analysed for their mRNA expression levels in this study.

The degenerate primers were designed with a focus on conserved regions in order to reduce degeneracy. This technique is cost effective and promising for designing primers for new species with no available information. However, this could fail to amplify if the degeneracy level is too high. Therefore, we could get successful amplification of only few genes. Sanger sequencing of the amplified products from degenerate PCR not only confirmed the identity of the genes but also provided the sequence for designing real time PCR primers.

The selected target genes for mRNA expression were found as differentially expressed proteins and/or mRNA in studies focusing on stress or pathogens exposure^15,49,56^. In the present study these genes are found to be expressed under normal physiological conditions. We have not compared the expression levels of these genes upon exposure to pathogen/stressor, however we have studied the expression levels in the three regions in lumpfish skin. The fact that these genes (*apoa1*, *hspa8*, *calm*, *hist1h2b*) show differences in expression between regions provide baseline knowledge, that should be kept in mind when comparing results from different studies, since skin sampling area makes a difference. The *apoa1* and *hist1h2b* exhibit antimicrobial properties and the *calm* and *hspa8* have roles in immune and/or stress response in fish^21–23,49^. The proteins encoded from the four target genes were identified in the skin of lumpfish in this study (Table S1) and in skin mucus in our previous lumpfish study^16^. Among the selected target genes the mRNA expression pattern did not follow the protein expression pattern. Similar results showing no correlation between the mRNA and protein expressions were observed in Atlantic cod challenged with *V*. *anguillarum*^62^ and in yeast (*Saccharomyces cerevisiae*)^63^ where the proteomic data did not correlate with the gene expression data. There are a number of factors that affects the mRNA-protein correlation such as the secondary structure of mRNA which changes continuously or under certain conditions affecting the translation efficiency, regulatory proteins could repress translation, codon bias and ribosomal density affects the translation of proteins, protein half lives after post translational modifications also serves as a major factor influencing the mRNA-protein correlation^64^. There are also other studies that concluded that the correlation between protein and mRNA expression often is very poor^65,66^. Changes in protein expression is not directly influenced by gene expression, and translational regulations have higher influence on the protein abundance than protein turnover^66^.

Alcian blue and Periodic acid Schiff stained skin sections of lumpfish observed by light microscopy showed thicker epidermis in the ventral region than the dorsal and caudal regions. Similar results were observed in a study conducted on gilthead sea bream (*Sparus aurata*) with thicker epidermis in ventral region than dorsal^9^. In benthic species the ventral epidermis is often thicker than other regions^67^. Thicker ventral epidermis in lumpfish could be due to the sedentary nature of the fish while adhering to the substrates in its habitat. Lumpfish skin sections showed a dermal pigment deposition in dorsal and caudal regions but not in the ventral region. Pigment cells have been seen in many teleost species (both scale and scaleless) in the dermal layer^68^. We identified several cells similar to saccular cells in Atlantic halibut^69^. These cells appeared as single vacuole in the epidermis in dorsal and caudal regions only. These cells did not respond to Alcian blue (pH-2.5) stain as suggested by Mittal *et al*.^70^ and Ottesen *et al*.^69^. Goblet cells (mucus producing cells) are important features of fish epidermis and are found in most of the teleost species. These cells vary in numbers depending on the location in the body, sex, life stages and physiological conditions such as infection^1^. We observed a relatively higher numbers of goblet cells in the ventral region than the dorsal and caudal regions. This could be to produce more mucus to provide a protective layer against invaders in the area where the fish is attached to surrounding objects by its adhesive disc. The high number of goblet cells in the ventral region suggests a higher synthesis of mucus proteins in this region, which correlates to the proteomics findings in the present study where relatively high expression of proteins were observed in the ventral region compared to the other two regions. However, due to unavailability of specific antibodies against the proteins, no obvious link was possible to establish between histological results and the molecular findings in this study. A connection between differences in goblet cell density and expression of goblet cell specific genes/protein could possibly be made when the genomic and transcriptomic resources become available for lumpfish. Nonetheless, this study provides useful information on skin molecular and structural parameters of the scorpaeniform lumpfish that would serve as a starting point to study the biology of this species.

## Conclusion

In the present study we used 2D gel based proteomics and LC-MS/MS to identify skin proteins of lumpfish, seventeen of these were differentially expressed among the dorsal, caudal and ventral regions of lumpfish skin. Using light microscopy we observed structural differences among the three regions of skin in terms of epidermal thickness, goblet cell counts and saccular cells. The epidermal thickness and goblet cell count were relatively higher in ventral region than in the other two regions. We did not observe saccular cells in ventral region. This is the first study to report differences of protein expression among different parts of skin in fish. It could provide a platform for quantitative comparison of skin proteome under various physiological conditions focusing on specific body sites. All together this study provides a sound knowledge about lumpfish skin structure and its associated molecular factors.

## Materials and Methods

### Fish rearing and tissue sampling

Lumpfish larvae (2 days post hatching) were obtained from Arctic Cleanerfish, Stamsund, Norway and reared at Mørkvedbukta Research Station, Nord University, Bodø, Norway. Larvae were raised in 80 l capacity black circular tanks with flow through seawater at 10–12 °C. All fish were fed with commercial diet from Skretting, Norway. Lumpfish (*n* = 6) of approximate weight 60–70 g were anaesthetised in MS-222 (70 mg/l) and humanely killed by giving a blow to the head. Skin samples from three different regions (D, C, V) were excised using a clean, sterile scalpel. For proteomic analysis the tissue pieces (~5 mm^2^) were snap frozen in liquid nitrogen and for real time PCR the tissue pieces of ~2 mm^2^ were submerged in 5 volumes of RNA later (25 mM sodium citrate, 20 mM EDTA, 70% ammonium sulphate). All samples for proteomics and real time PCR were moved to −80 °C until further analysis. Skin tissues for histology were fixed in 10% phosphate buffered formaldehyde solution. All animal rearing and handling procedures were performed in accordance with national guidelines enforced by The Norwegian Food Safety Authority (www.mattilsynet.no/language/english/about_us/). Under local legislation, an ethical approval is not needed for experiments where animals are only killed to take organs or tissues, as it was done in the present study. The skin samples used in this study were sampled along with the mucus. However, the amount of mucus protein/RNA from a tissue of size ~5 mm^2^/~2 mm^2^ used in this study is negligible compared to the protein/RNA extracted from skin. In our experience lumpfish yields very low amount of skin mucus (a lumpfish weighing ~700 g yields 80–110 μg of mucus proteins when scraped from the whole body, unpublished data).

### Two-dimensional gel electrophoresis

Frozen skin samples were homogenised with liquid nitrogen by using pestle and mortar. Homogenized tissue was mixed with 1x PBS (1 ml of 1xPBS for homogenized powder yielded from 5 mm^2^ of tissue) containing 0.01% of 100x protease inhibitor (GE Healthcare Life Sciences) and sonicated two times (5 s each with an interval of 1 min) on ice using an ultrasonic processor (SONICS Vibracell VCX750, USA). The sonicated sample was centrifuged at 15,000 g for 30 min at 4 °C to pellet the debris in the tissue sample. The resulted supernatant was processed as described in^16^. Protein was quantified using Qubit Fluorometer, Invitrogen. IPG strips (pH 3–10, 17 cm, BioRad, USA) were rehydrated with 100 μg of protein and electro focused. Electro focused strips were run on 12.5% polyacrylamide gels for approximately 16 h and stained in SYPRO^®^ Ruby (ThermoFisher Scientific, USA) fluorescent protein stain. Gel images were documented using ChemiDoc^®^ XRS system (BioRad, USA) and used for PDQuest (BioRad, USA) analysis.

### LC-MS/MS and protein identification

Abundant and/or spots with different expression levels among three regions (D, C, V) were excised and subjected for LC-MS/MS analysis. LC-MS/MS analysis was performed at University of Tromsø, Norway by using nanoACQUITY ultra performance liquid chromatography system and Q-TOF mass spectrophotometer (Micromass/Waters, MA, USA). The peak list files generated from LC-MS/MS analysis were analysed by MASCOT MS/MS Ion search (version 2.5). A homology driven search was performed using various protein databases including SwissProt (553941 sequences; 198311666 residues, March 2017) and NCBInr (116205035 sequences; 42603624384 residues, March 2017) for protein identification. Parameters set for identification were carbamidomethyl (C) fixed modification, oxidation (M) variable modification, monoisotopic, peptide charge 2+ and 3+, enzyme trypsin with maximum 1 missed cleavage, peptide tolerance 100 ppm and MS/MS tolerance 0.1 Da. The identification was restricted to taxonomic group Actinopterygii keeping the false discovery rate below 1%. Proteins showing significant hits (p < 0.05) with a score above threshold level and at least one unique peptide sequence were identified.

### Gene ontology and protein-protein interaction

Gene ontology terms of identified proteins were retrieved from their human orthologues from UniProt KB protein database according to their biological process. The protein-protein interaction map was constructed using string v.10 (www.string-db.org) with a high edge confidence limit. Zebrafish orthologues of identified proteins were used as input for protein protein interaction analysis due to unavailability of species-specific data for lumpfish. String asigns the names for the proteins as they are found in the databases (Biocarta, BioCyc, GO, KEGG and Reactome) from where string extracts the curated data, thus the actual names of proteins identified varies from the names in the interaction network. The original names and their corresponding string names are mentioned in Supplementary Table S2. The string analysis was performed with high confidence limit (<0.700, maximum confidence limit is 1), the thicker the joining lines are, the stronger the interactions are. The protein interaction sources were obtained from text mining, experiment, databases, co-expression, neighbourhood and co-occurrence. The proteins in the map are shown by nodes and connections are shown by edges. Nodes represent proteins produced by a single protein coding gene locus. Colored nodes are first shell interactors and white shells are second cell interactors in the protein interaction network. Filled nodes show that the 3D structure of the protein is known and empty nodes shown that the structure is unknown. The edges represent association of proteins, that jointly could contribute to a shared function, they do not necessarily have to bind to each other physically.

### RNA extraction and cDNA preparation

Total RNA from lumpfish skin was extracted by using E.Z.N.A.^®^ Total RNA Kit (Omega Bio-tek, Norcross, GA) following the manufacturer’s protocol. RNA integrity was determined by observing two distinct bands representing 18S and 28S on 1% agarose gel. RNA was quantified using Qubit RNA BR assay kit and the Qubit Fluorometer (ThermoFisher Scientific, USA). The extracted RNA was reverse transcribed to synthesize cDNA from 1 μg of total RNA using QuantiTect reverse transcription kit (Qiagen, Germany) as described by the manufacturer. The cDNA samples were 50x diluted for qPCR analysis.

### Primer design

Till date lumpfish genome has not been sequenced and also the nucleotide sequences of the selected proteins are not available in the databases. Therefore, degenerate primers were designed using geneious9 software (Biomatters, New Zealand) and restriction sites (GCTGGCGCCTCTCTAGACACAGGATCC for forward and GTCGACAAGGGTACCATAGAAGGGAGAAGC for reverse) were added to each primer. PCR amplification for degenerate primers were performed under the following conditions: initial denaturation at 94 °C for 2 min, followed by 34 cycles of 94 °C for 30 s, 50 °C for 30 s, 72 °C for 2 min, and final extension at 72 °C for 2 min. The PCR products were ran on 1% agarose gel. Expected bands from the gel were excised and DNA was purified using NucleoSpin^®^ Gel and PCR Clean-up (Macherey-Nagel, Germany). Purity and concentration of gel purified DNA was analysed by Nanodrop 1000, (ThermoFisher Scientific, USA). Further, the purified DNA was sequenced using ABI 3100 DNA sequencer, (Applied Biosystems, USA) using Big dye termination chemistry (ThermoFisher Scientific, USA). Sequences obtained from the DNA sequencer were used for real time primer designing. The oligonucleotide sequences and specifications are mentioned in Table 1. All primers used for real time analysis were sequenced and blasted against NCBI to confirm their identification.

### Quantitative real time PCR

Three reference genes *gapdh*, *ef1-alfa* and *β-actin* were selected for the study. The Excel based tool BestKeeper^71^ was used to analyse stability of the genes. Real time quantitative PCR was performed on Applied Biosystems Step OnePlus using SYBR green chemistry (Applied Biosystems, USA).

Standards were prepared to generate calibration curve for estimation of PCR efficiency of primers. Total RNA samples were pooled and reverse transcribed to make cDNA for preparation of standards. Five series of three fold dilutions (1:3, 1:9, 1:27, 1:81, 1:243, 1:729) were prepared from undiluted cDNA. The cDNA from each dilution was further diluted to 1:3 dilutions with molecular grade water. PCR efficiency (E) for each primer was calculated according to formula E = 10^(−1/slope)^72^.

All gene amplifications were performed in a total volume of 10 μl containing 5 μl of SYBR^®^ green PCR master mix, 4 μl of template DNA and 1 μl of primer mix (5 μM of each forward and reverse primers). The amplification condition involves a holding stage for 20 s at 95 °C followed by 35 cycles of denaturation at 95 °C for 3 s, annealing at 60 °C, for 30 s and during each cycle at the annealing stage data acquisition step was included for 15 s at 60 °C. All plates were run with negative controls (no template control and no reverse transcriptase control) and positive control (pooled cDNA from all samples). All reactions were carried out in triplicates. The qPCR data was checked for normality using Shapiro Wilks normality test and homogeneity of variance was checked using Bartlett test. The data was analysed by employing one-way ANOVA (analysis of variance) followed by Tukey HSD (honest significant difference) post hoc analysis (p < 0.05, *n* = 6). All the analysis were performed in GraphPad Prism7 software (https://www.graphpad.com/).

### Histology

Lumpfish skin tissues of approximately 0.5 cm^2^ from different regions (D, C, V) were sampled (*n* = 5) using a clean, sterile scalpel, immediately fixed in 10% phosphate buffered formaldehyde solution and left at room temperature for 24 h. The samples were dehydrated using a standard histological technique with a series of graded ethanol treatments, embedded in paraffin and sectioned into 4 μm sections. Skin sections were stained with a combination of 1% Alcian blue and Periodic acid Schiff (pH 2.5) stain. Photomicrographs of skin sections were prepared using light microscopy and Cell B imaging software (Olympus, Germany). Goblet cells were counted for each region from five fishes. For goblet cell counting three equal sized area (0.06 mm^2^) from each section were randomly selected and cells were counted using manual settings in Fiji software v2.0.0 (https://fiji.sc). Thickness of skin epidermis of the three regions was measured. Both thickness measurements and goblet cell counts were statistically analysed using ANOVA and Tukey HSD post hoc analysis (p < 0.05).

## Supplementary information

Proteomic and structural differences in lumpfish skin among the dorsal, caudal and ventral regions

## Data Availability

The datasets generated and analysed during the current study are available from the corresponding author on reasonable request.
