# Supplementary material for: Proteomic and structural differences in lumpfish skin among the dorsal, caudal and ventral regions
Source: Sci Rep. 2019 May 6;9:6990. doi: 10.1038/s41598-019-43396-z (PMC6502863; doi:10.1038/s41598-019-43396-z)

Title: Proteomic and structural differences in lumpfish skin among the dorsal, caudal and ventral regions

Deepti M. Patel[1,2], Katarina Bhide[2], Mangesh Bhide[2], Martin H. Iversen[1] and Monica F. Brinchmann[1*]

[1]Faculty of Biosciences and Aquaculture, Nord University, 8049 Bodø, Norway.
[2]Laboratory of Biomedical Microbiology and Immunology, 73, 04181, University of Veterinary Medicine and Pharmacy, Košice, Slovakia.

*Corresponding author
monica.f.brinchmann@nord.no

Supplementary Table S1. Lumpfish skin proteins identified by MASCOT

Spot ID	Protein name (Species)	Accession number	Apparent PI/MW	Significant threshold/ p-value	Protein score/Up	SC (%)	Peptide sequence	GO terms	Data base
D2	Prefoldin subunit 1 (*Danio rerio*)	Q5D016; A7MCD8	8.83/13978	21/p<0.05	45/45	9	KLADLQIEQLSRV	protein folding (GO:0006457)	Swiss prot
D3	Glial fibrillary acidic protein (*Carassius auratus*)	P48677	4.93/42636	20/ p<0.05	28/28	3	KLALDIEIATYRK	cytoskeleton organization (GO:0007010)	Swiss prot
D5	40S ribosomal protein S12 (*Oreochromis niloticus*)	O13019	6.30/14726	20/ p<0.045	159/159	24	KDVIEEYFKS<br>KLGEWVGLCKI<br>KLVEALCAEHQINLIKV	rRNA processing (GO:0006364)	Swiss prot
D6	Glial fibrillary acidic protein (*Carassius auratus*)	P48677	4.93/42636	20/ p<0.05	41/41	3	KLALDIEIATYRK	cytoskeleton organization (GO:0007010)	Swiss Prot
D8	Fatty acid binding protein (*Salvelinus fontinalis*)	AIK01712	5.77/8299	49/ p<0.037	62/62	10	LGVGFATRQ	epidermis development (GO:0008544)	NCBI
D9	Ubiquitin-40S ribosomal protein (*Ictalurus punctatus*)	P68200; P68199; Q90YP4	9.68/18330	20/ p<0.049	135/135	17	KEGIPPDQQRL<br>KESTLHLVLRL<br>RTLSDYNIQKE	nucleotide excision repair, DNA damage recognition (GO:0000715)	Swiss Prot
D10	Hemoglobin subunit beta (*Cottoperca gobio*)	P84652	6.96/16481	20/ p<0.05	50/50	12	MVEWTDFERA<br>KFLAVVVSSLGRQ	transport (GO:0006810)	Swiss prot
D11	Hemoglobin subunit beta-2 (*Pseudaphritis urvillii*)	P83625	5.93/16443	21/ p<0.045	176/138	26	RATIKDIFSKI<br>VEWTDFERA<br>KFLAVVVSSLGRQ<br>RCLVVYPWTQRY	transport (GO:0006810)	Swiss Prot
D12	Parvalbumin beta-2 (*Theragra chalcogramma*)	Q90YK7	4.60/11614	22/ p<0.035	47/47	17	KLFLQNFSASARA	calcium ion binding (GO:0005509)	Swiss Prot
D13	Lipocalin-like (*Xiphophorus maculatus*)	XP_00580 3374	4.94/21354	50/ p<0.03	118/118	5	KDGVSEVLNKL<br>KTKDGVSEVLNKL	Lipid metabolic process (GO:0006629)	NCBI
D14	Collagen alpha1 (I) chain like isoform X1 (*Hippocamus comes*)	XP_01974 0420	5.72/138015	48/p<0.05	129/129	1	RTGGSCTLDGQVFADRD	skeletal system development (GO:0001501)	NCBI
D15	Natterin-2 (*Thalassophryne nattereri*)	Q66S21	8.90/41985	21/ p<0.049	28/28	2	KADIPFTATLIRT	NA	Swiss Prot
D16	Keratin, type II cytoskeletal 8 (*Danio rerio*)	Q6NWF6; Q7ZT78	5.15/57780	23/ p<0.03	39/39	1	RFASFIDKV	keratinization (GO:0031424), extrinsic	Swiss Prot

								apoptotic signaling pathway (GO:0097191)	
D17	Collagen alpha-2(I) chain isoform X1 (*Stegastes partitus*)	XP_008287498	9.41/127388	51/p<0.02	535/154	4	RAKDYEVDATIK.S<br>KAVVLQGSNDVELRA<br>RFTFSVLEDGCTRH<br>KKAVVLQGSNDVELRA<br>RLPLLDIAPLDIGGADQEF<br>GLDIGPVCFK	skeletal system development (GO:0001501)	Swiss Prot
D18	Calmodulin (*Ctenopharyngodon idella*)	Q6IT78	4.09/16827	25/p<0.019	25/25	10	KELGTVMRS<br>KDTDSEEEIRE	calcium ion binding (GO:0005509)	Swiss Prot
D20	60S ribosomal protein L30 (*Ictalurus punctatus*)	P58372	9.65/13105	20/p<0.05	91/91	24	KLVILANNCPALRK<br>RVCTLAIIDPGDSDIIRS	translation (GO:0006412)	Swiss Prot
D21	Histone H2A (*Danio rerio)*	Q71PD7	10.58/13501	20/p<0.05	55/55	14	RAGLQFPVGRI<br>RGDEELDSLIKA	cellular response to DNA damage stimulus (GO:0006974)	Swiss Prot
D22	Fatty acid-binding protein (*Maylandia zebra*)	XP_004549259	6.34/15269	49/p<0.04	208/133	23	KAIGVGFATRQ<br>KCIMGDVIAVRT<br>KLNEPFDETTADDRK	epidermis development (GO:0008544)	NCBI
D23	Histone H2B (*Danio rerio*)	Q5BJA5	10.37/13569	20/p<0.05	64/64	12	REIQTAVRL<br>RLLLPGELAKH	DNA binding (GO:0003677), protein heterodimerization activity (GO:0046982)	Swiss Prot
D24	Histone H4 (*Oncorhynchus mykiss*)	P62797; P02304; P02305	11.36/11360	21/p<0.05	24/24	7	KVFLENVIRD	nucleosome assembly (GO:0006334)	Swiss Prot
D25	Parvalbumin beta (*Scomber japonicus*)	P59747; Q7ZW61	5.15/11652	22/p<0.035	52/52	16	KSGFIEEEELKL	calcium ion binding (GO:0005509)	Swiss Prot
D26	Myotrophin (*Takifugu rubripes*)	XP_003972762	4.96/12996	52/p<0.05	53/53	7	KLVTAEDVNRT	cellular response to mechanical stimulus (GO:0071260)	NCBI
D27	unnamed protein product (Tetraodon nigroviridis)	CAG11620	5.96/18450	59/p<0.0035	60/60	7	KALAAGGVGSIVRV	NA	NCBI
D28	Ubiquitin-conjugating enzyme E2 (*Danio rerio*)	Q6PEH5	7.79/16370	28/p<0.0047	68/68	6	RLLEELEEGQKG	cellular response to DNA damage stimulus (GO:0006974)	Swiss Prot

D29	Myosin light chain 2, isoform B (*Hippoglossus hippoglossus*)	CAD32552	4.72/19213	54/p<0.013	154/154	14	KGADPEDVIITAFKV<br>KVLDPEATGSIKK	muscle contraction (GO:0006936)	NCBI
D30	Apolipoprotein A1 (*Xiphophorus maculatus*)	XP_005799476	4.68/28589	49/p<0.045	122/122	7	KVQVELTQRA<br>KDLQAQLGPYTDDLKQ	lipoprotein metabolic process (GO:0042157)	NCBI
D31	Apolipoprotein A1, partial (*Morone saxatilis*)	ACH90227	4.75/20591	58/p<0.004	94/94	7	KALDQLDDTEYKELKA	lipoprotein metabolic process (GO:0042157)	NCBI
D32	Apolipoprotein A1, partial (*Morone saxatilis*)	ACH90227	4.75/20591	53/p<0.015	78/78	6	KALDQLDDTEYKE	lipoprotein metabolic process (GO:0042157)	NCBI
D33	Protein AMBP (*Pleuronectes platessa*)	P36992	5.30/40499	24/p<0.05	45/45	2	RDTVLDDFKT	cell adhesion (GO:0007155)	Swiss Prot
D34	40S ribosomal protein S3 (*Ictalurus punctatus*)	90YS2	9.8/27044	20/p<0.04	41/41	3	RTEIIILATRT	nuclear transcribed mRNA metabolic process (GO:0000184)	Swiss Prot
D35	Collagen alpha-2(I) chain (*Oncorhynchus mykiss*)	_	9.36/127364	21/p<0.035	249/249	1	KAVLLQGSNDVELRA<br>KKAVLLQGSNDVELRA	skeletal system development(GO:0001501)	Swiss Prot
D36	Actin, cytoplasmic 1 (*Ctenopharyngodon idella*)	83751; O73815; P12714	5.30/42068	30/p<0.005	47/47	1	KIIAPPERK	ATP dependent chromation remodelling (GO:0043044)	Swiss Prot
D37	Transcriptional activator protein Pur-beta (*Danio rerio*)	Q6PHK6; Q6NW99	5.53/32586	20/p<0.05	34/34	3	KIAEVGAGGSKS	transcription (GO:0006351)	
D38	Actin, alpha cardiac (*Takifugu rubripes*)	P53480	5.22/42290	20/p<0.05	988/381	54	KIIAPPERK<br>KRGILTLKY<br>KAGFAGDDAPRA<br>RDLTDYLMKI<br>RGYSFVTTAERE<br>KEITALAPSTMKI<br>KDSYVGDEAQSKR<br>RAVFPSIVGRPRH<br>RHQGVMVGMGQKD<br>KQEYDEAGPSIVHRK<br>KIWHHTFYNELRV<br>KSYELPDGQVITIGNERF<br>RVAPEEHPTLLTEAPLNPKA	actin filament based movement (GO:0030048), apoptotic process (GO:0006915)	Swiss Prot

							KYPIEHGIITNWDDMEKI RKDLYANNVLSGGTTMY PGIADRM KLCYVALDFENEMATAA SSSSLEKS		
D39	Hemoglobin subunit beta-1 (*Liparis tunicatus*)	P85082	6.64/16660	25/p<0.022	212/176	23	KLHVDPDNFKL KAFTGEVQAALQKF KLISDCLTIVVASRL	transport (GO:0006810)	Swiss Prot
D40	Hemoglobin subunit beta-1 (*Liparis tunicatus*)	P85082	6.64/16660	20/p<0.05	81/81	17	KAFTGEVQAALQKF KLISDCLTIVVASRL	transport (GO:0006810)	Swiss Prot
D41	Actin cytoplasmic 1 (*Ctenopharyngodon idella*)	P83751; O73815; P12714	5.30/42068	20/p<0.05	76/54	9	KDSYVGDEAQSKR KLCYVALDFEQEMGTAA SSSSLEKS	ATP dependent chromation remodelling (GO:0043044)	Swiss Prot
D42	Heat shock cognate 71 kDa (*Oryzias latipes*)	Q9W6Y1	5.80/76577	24/p<0.02	188/112	5	R.GTLDPVEK.S R.TTPSYVAFTDTER.L R.IINEPTAAAIAYGLDK.K RIINEPTAAAIAYGLDKK V	cellular response to stress (GO:1900034, GO:0009267), chaperone activity (GO:0061684, GO:0051085)	Swiss Prot
D43	Transferrin (*Trachidermus fasciatus*)	AEV2197 1	5.80/75092	49/p<0.03	438/130	7	KANYELLCKD KHLTVPESEKA KQTGDCDFTKF KSSGLTWETLKG KEADAMAVDGGQVYTA GKC RKEADAMAVDGGQVYT AGKC	cellular iron ion homeostasis (GO:0006879)	NCBI
D44	Serotransferrin (*Paralichthys olivaceus*)	O93429	6.06/76545	20/p<0.05	59/59	2	KEADAMAVDGGQVYTA GKC	cellular iron ion homeostasis (GO:0006879)	Swiss Prot
D45	Triosephosphate isomerase B (*Danio rerio*)	Q90XG0; Q7T315	6.45/27096	28/p<0.015	261/261	25	KFFVGGNWKM KGAFTGEISPAMIKD KTASPQQAQEVHDKL RHVFGESDELIGQKV KVVLAYEPVWAIGTGKT	glucose metabolic process (GO:0006006)	Swiss Prot
D46	Adenylate kinase isoenzyme 1 (*Cyprinus carpio*)	P12115	6.64/21532	21/p<0.05	171/171	26	RSDDNEETIKK KGYLIDGYPRE KATEPVIAYYETRG KQLQAIMQKG	ATP metabolic process (GO:0046034)	Swiss Prot

							KGELVPLDTVLDMIKD		
D47	Protein disulfide-isomerase precursor (*Ictalurus punctatus*)	JZ585147	5.17/31098	57/p<0.04	252/72	18	KSNQLPLVIEFTEQTAPKI<br>KSNQLPLVIEFTEQTAPKI<br>KGKILFIFIDSDVDDNQRI	cell redox homeostasis (GO:0045454)	NCBI
D48	Protein disulfide-isomerase (*Maylandia zebra*)	XP_004538825	4.57/57378	47/p<0.05	77/77	3	KVDATEETELAQEYGVRG	cell redox homeostasis (GO:0045454)	NCBI
D50	Cofilin-1 (*Onchorynchus mykiss*)	ACO07669	8.48/18784	50/p<0.03	87/87	6	RYALYDATYETKE	actin cytoskeleton organization (GO:0030036)	NCBI
D52	Transcription factor BTF3 homolog 4 (*Danio rerio*)	Q6PC91	5.95/17350	21/p<0.05	29/29	5	KLAEQFPRQ	NA	Swiss Prot
D55	40S ribosomal protein S25 (*Danio rerio*)	Q6PBI5	10.14/13852	20/p<0.05	109/109	16	KLITPAVVSERL<br>RDKLNNLVLFDKA	nuclear transcribed mRNA metabolic process (GO:0000184)	Swiss Prot
D56	ATP synthase subunit d, mitochondrial-like (*Lates calcarifer*)	XP_018535231	7.85/18181	48/p<0.05	118/118	11	KSASAYIEGSKA<br>KAGMVDEFEKK	ATP biosynthetic process (GO:0006754)	NCBI
D57	Proteasome subunit alpha type-5, partial (*Larimichthys crocea*)	KKF13888	4.77/25745	48/p<0.054	419/419	29	RGVNTFSPEGRL<br>RLFQVEYAIEAIKL<br>RITSPLMEPNSIEKI<br>KLNATNIELATVEPGKT<br>RAIGSASEGAQSSLQEVYHKS	Post translational protein modification(GO:0043687), proteosomal protein catabolic process (GO:0010498)	NCBI
D59	Cofilin-2 (*Onchorynchus mykiss*)	ACO07669	8.84/18784	48/p<0.05	83/83	6	RYALYDATYETKE	actin filament depolymerisation (GO:0030042)	NCBI
D60	Unnamed protein product, partial (*Tetraodon nigroviridis*)	CAG09787	6.82/18856	52/p<0.025	146/146	14	RYALYDATYETKE<br>KVTDEVIAVFNDMKV	NA	NCBI
D62	60S acidic ribosomal protein P0 (*Danio rerio*)	Q9PV90	6.16/34902	21/p<0.05	40/40	6	RGNVGFVFTKE	translation (GO:0006412)	Swiss Prot
D63	Guanine nucleotide-binding protein subunit beta-2-like 1 (*Oreochromis niloticus*)	O42249	8.07/35541	28/p<0.01	570/570	35	KIIVDELRQ<br>KIWDLEGKI<br>RQEVISTNSKA<br>RVWQVTIGTR<br>KLWNTLGVCKY<br>RDETNYGIPQRS	activation of adenylate kinase activity (GO:0007190), cellular response to catecholamine stimulus (GO:0071870)	Swiss Prot

							KDVLSVAFSADNRQ<br>RYWLCAATGPSIKI<br>KDGQAMLWDLNEGKH<br>KGHSGWVTQIATTPKYP<br>DMILSASRD		
D64	Guanine nucleotide-binding protein subunit beta-2-like 1 (*Oreochromis niloticus*)	O42249	8.07/35541	20/p<0.025	381/381	37	KIIVDELRQ<br>KIWDLEGKI<br>RVWQVTIGTR<br>RDETNYGIPQRS<br>KDVLSVAFSADNRQ<br>RYWLCAATGPSIKI<br>KDGQAMLWDLNEGKH<br>KIIVDELRQEVISTNSKA	activation of adenylate kinase activity (GO:0007190), cellular response to catecholamine stimulus (GO:0071870)	Swiss Prot
D65<br>D66	60S acidic ribosomal protein P0 (*Danio rerio*)	Q9PV90	6.16/34902	21/p<0.05	108/108	6	RGNVGFVFTKE<br>KTSFFQALGITTKI	translation (GO:0006412)	Swiss Prot
D67	costars family protein ABRACL (*Larimichthys crocea*)	XP_01073 7667	5.66/9091	56/p<0.007	55/55	9	KLLVEEIQRL	NA	NCBI
D68	Parvalbumin-2 (*Danio rerio*)	Q9I8V0; Q567L1	4.46/11672	20/p<0.05	147/147	20	KIGVDEFALLVKA<br>KLFLQNFSAGARA	regulation of calcium ion concentration (GO:0051480)	Swiss Prot
D69<br>D70	Elongation factor 1-alpha (*Danio rerio*)	Q92005	9.16/50301	21/p<0.04	47/47	2	KIGGIGTVPVGRV	transcription (GO:0006351), cellular response to epidermal growth factor stimulus (GO:0071364)	Swiss Prot
D71	ATP synthase subunit beta, mitochondrial (*Cyprinus carpio*)	Q9PTY0	5.05/55327	28/p<0.01	709/709	28	KVLDTGAPIRI<br>KIGLFGGAGVGKT<br>RIPVGPETLGRI<br>KVVDLLAPYAKG<br>RTIAMDGTEGLVRG<br>RIMNVIGEPIDERG<br>RFTQAGSEVSALLGRI<br>KTVLIMELINNVAKA<br>K.VALVYGQMNEPPGAR.<br>A<br>RDQEGQDVLLFIDNIFRF	ATP biosynthetic process (GO:0006754)	Swiss Prot

							REGNDLYHEMIESGVINL KD KSLQDIIAILGMDELSEED KLTVARA		
D72	Tubulin beta-1 chain (*Gadus morhua*)	Q9YHC3	4.79/50173	31/0.004	1063/212	35	KTAVCDIPPRG RYLTVAAIFRG KNMMAACDPRH RFPGQLNADLRK KLAVNMVPFPRL RISEQFTAMFRR RKLAVNMVPFPRL RINVYYNEASGGKY RIMNTFSVVPSPKV KEVDEQMLNVQNKN RLHFFMPGFAPLTSRG REIVHLQAGQCGNQIGAK F KMAATFIGNSTAIQELFK R KGHYTEGAELVDSVLDV VRK	spindle assembly (GO:0051225)	Swiss Prot
D73	Keratin, type II cytoskeletal 8 (*Danio rerio*)	Q6NWF6; Q7ZT78;	5.15/57780	30/p<0.006	333/333	8	RFASFIDKV RFLEQQNKM KLLEGEEDRL KYEDEINKRT RSNIDAMFEAYIANLRR	keratinization (GO:0031424), extrinsic apoptotic signaling pathway (GO:0097191)	Swiss Prot
D74	Keratin, type II cytoskeletal 8 (*Danio rerio*)	Q6NWF6; Q7ZT78	5.15/57780	27/p<0.008	306/306	11	RFASFIDKV RFLEQQNKM KLLEGEEDRL RAQYEDIANRS KLESLTDEINFLRQ KLEADLHNMQGLVEDFK N	keratinization (GO:0031424), extrinsic apoptotic signaling pathway (GO:0097191)	Swiss Prot
D75	Glyceraldehyde-3-phosphate dehydrogenase 2 (*Danio rerio*)	Q5MJ86	6.55/36426	22/p<0.03	167/167	12	RGAHQNIIPASTGAAKA RVPVADVSVVDLTCRL	Glycolytic pathway (GO:0006096), glucose metabolic process (GO:0006006)	Swiss Prot

D76	Protein AMBP (*Pleuronectes platessa*)	P36992	5.30/40499	20/p<0.05	40/40	2	RDTVLDDFKT	cell adhesion (GO:0007155)	Swiss Prot
D78	60S ribosomal protein L18 (*Salmo salar*)	P24558	11.94/20563	21/p<0.05	25/25	4	KSVLLSAPRN	rRNA processing (GO:0006364)	Swiss Prot
D80	Rhophilin-2 (*Danio rerio*)	Q6TNR1; Q803B1	7.55/77692	20/p<0.05	35/35	1	KAEMEIPAATKV	signal transduction (GO:0007165)	Swiss Prot
D82	Tropomyosin alpha-1 chain (*Danio rerio*)	P13104	4.70/32760	25/p<0.02	246/246	16	KLDKENALDRA RIQLVEEELDRA KTIDDLEDELYAQKL KAISEELDHALNDMTSI	actin filament organization (GO:0007015)	Swiss Prot
D83	40S ribosomal protein SA (*Danio rerio*)	Q803F6	4.75/34162	23/p<0.03	232/94	19	RLLIVTDPRA RFTPGTFTNQIQAAFRE RAIVAIENPADVCVISSRN REHPWEVMPDLYFYRDP EEIEKE	cell adhesion (GO:0007155)	Swiss Prot
D84	Malate dehydrogenase 2-2, NAD (mitochondrial) (*Oreochromis niloticus*)	CDQ8417 6	8.15/35782	53/p<0.015	508/180	30	KYFSTPLLLGKH RVFGVTTLDIVRA RFTFSVLDAMNGKE KAGAGSATLSMAYAGAR F KVEFPADQLSALTARI KVAVLGASGGIGQPLSLL LKN RDDLFNTNATIVATLADA VARN	carbohydrate metabolic process (GO:0005975)	NCBI
D85	Annexin A1 (*Notothenia coriiceps*)	XP_01078 8362	6.50/37631	48/p<0.05	79/79	5	KGDLEEVVLALLKT	actin cytoskeleton organization (GO:0031532) , adaptive immune response (GO:0002250)	NCBI
D86	N-acylneuraminate cytidylyltransferase B (*Danio rerio*)	H9BFW7; E7F0X7	6.30/48034	21/p<0.05	22/22	2	RAALILARGGSKG	metabolic process (GO:0006054)	Swiss Prot
D88	Proteasome subunit alpha type-2 (*Carassius auratus*)	O73672	5.99/25918	21/p<0.05	120/120	15	KASNGVVLATEKK RGYSFSLTTFSPSGKL	Post translational protein modification(GO:0043687) , proteosomal protein	Swiss Prot

								catabolic process (GO:0010498)	
D89	Triosephosphate isomerase A (*Danio rerio*)	Q1MTI4; Q7ZWB0; Q90XF9	4.90/27179	20/p<0.05	38/38	5	RHVFGESDELIGQKV	glucose metabolic process (GO:0006006)	Swiss Prot
D91	COP9 signalosome complex subunit 2 (*Danio rerio*)	Q6IQT4	5.31/51824	24/p<0.025	28/28	1	KQMIKINFKL	negative regulation of transcription(GO:0000122)	Swiss Prot
D92	Elongation factor 1-delta (*Salmo salar*)	ACM08644	4.59/26363	48/p<0.05	58/58	7	KSSILLDVKPWDDETDMSKL	transcription (GO:0006351)	NCBI
D93	Myosin light chain 1, skeletal muscle isoform (*Liza ramada*)	P82159	4.54/20054	21/ p<0.05	89/89	11	RIVLSTLGEKM KEGNGTVMGAELRI	muscle contraction (GO:0006936)	Swiss Prot
D95	Collagen alpha-2(I) chain (*Larimichthys crocea*)	KKF26459	9.15/119089	55/p<0.0087	142/142	2	RFTFSVLEDGCTRH KSLNTQIENLLTPEGSRK	skeletal system development(GO:0001501)	NCBI
D96	Natural killer enhancing factor (*Scopthalmus maximus*)	ABF01135	5.58/22063	48/p<0.048	181/181	17	KIPIVADLTKT RQITINDLPVGRS RDYGVLKEDDGIAYRG	response to reactive oxygen species (GO:0000302)	NCBI
D97	Proteasome subunit beta type 6 (*Osmerus mordax*)	ACO09878	5.13/25238	52/p<0.02	64/64	4	RTTTGAYIANRV	Post translational protein modification(GO:0043687), proteosomal protein catabolic process (GO:0010498)	NCBI

PI; isoelectric point, MW; molecular weight, Up; total score of unique peptide, SC; sequence coverage, NA; data not available, GO terms; gene ontology term of biological process of the proteins retrieved manually from UniProt KB, Missing spot number signifies that those proteins were not identified. Unique peptides are marked in red letters and peptides in black letters are not unique.

Supplementary Table S2. Abbreviation of protein names used in protein interaction map in Fig. 4 (All abbreviations are assigned by string v.10)

Protein name	Abbreviation
Actin, heart	acta1b, actc1
Adenylate kinase isoenzyme 1	ak1
Annexin A1	zgc:110283
Apolipoprotein A1	apoa1a
ATP synthase subunit d	atp5h
Calmodulin	calm1a
Cofilin-1	cfl1
Cofilin-2	cfl2l
Collagen alpha-1	col1a1a
COP9 signalosome complex subunit 2	cops2
Costars family protein ABRACL	abracl
Elongation factor 1-alpha	eef1a2
Fatty acid binding protein	fabp3
Glial fibrillary acidic protein	gfap
Glyceraldehyde-3-phosphate dehydrogenase 2	gapdh
Guanine nucleotide-binding protein	gnb1a
Haemoglobin subunit beta	hbbe2
Heat shock cognate 71 kDa	hspa8
Histone H2A	loc560309
Histone H2B	cr762436.3
Histone H4	hist1h4l
Keratin cytoskeletal protein	krt8
Malate dehydrogenase	mdh2
Myotrophin	mtpn
Natural killer enhancing factor	prdx1
Parvalbumin beta-2	pvalb2
Prefoldin 1	pfdn1
Proteasome subunit alpha	psma2
Proteasome subunit beta	psmb1
Protein disulfide-isomerase	pdia2
Rhophilin-2	rhpn2
40S ribosomal protein SA	rpsa
40S ribosomal protein S25	rsp25
60S ribosomal protein L18	rpl18
60S acidic ribosomal protein P0	rplp0
Serotransferrin	tfa
Transcriptional activator protein Pur-beta	purb
Transcription factor BTF3 homolog 4	btf3l4
Transferrin	tfa
Triosephosphate isomerase A	tpi1a
Triosephosphate isomerase B	tpi1b

Tropomyosin alpha-1	tpma
Tubulin beta-1	tubb5
Ubiquitin conjugating enzyme E2	ube2v2

Supplementary Table S3. BestKeeper analysis details of three reference genes *gapdh, ef1-alfa, β-actin*

**Gene name**	***gapdh***	***ef1-alfa***	***β-actin***
**n**	18	18	18
**SD [± Cq]**	0.63	0.52	0.71
**CV [% Cq]**	2.68	2.49	3.64
**r**	0.930	0.882	0.865
**r^2**	0.865	0.778	0.748
**p value**	0.001	0.001	0.001
**Ranking**	1	2	3

n; number of samples, SD [± Cq]; standard deviation of Cq values, CV [% Cq]; co-efficient of variation as % of Cq values, r; co-efficient of correlation, r^2; co-efficient of determination.

Supplementary Figure 1. Images of 2D gels used for analysis in this study.
Figure D1-D6 are gels from the dorsal region, C1-C6 are gels from caudal region and V1-V6 are gels from the ventral region.

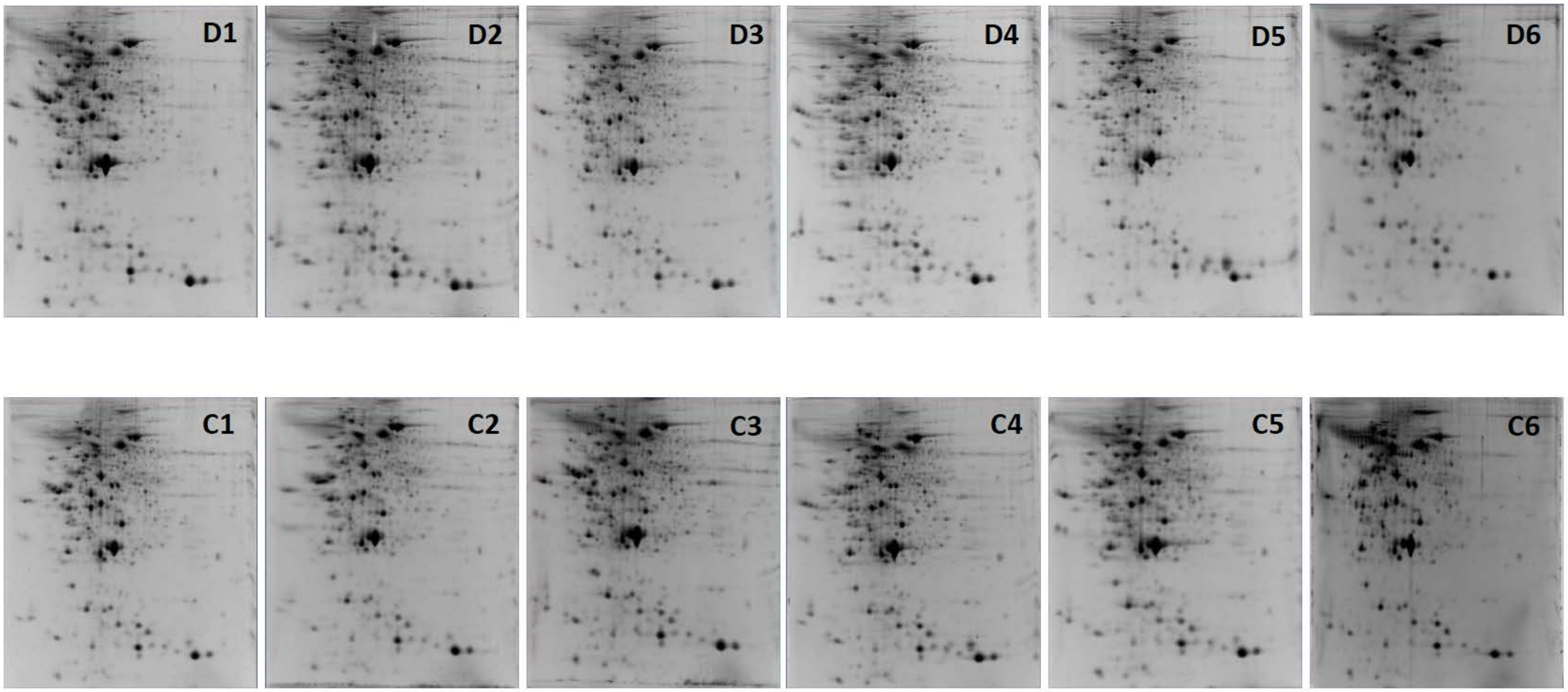

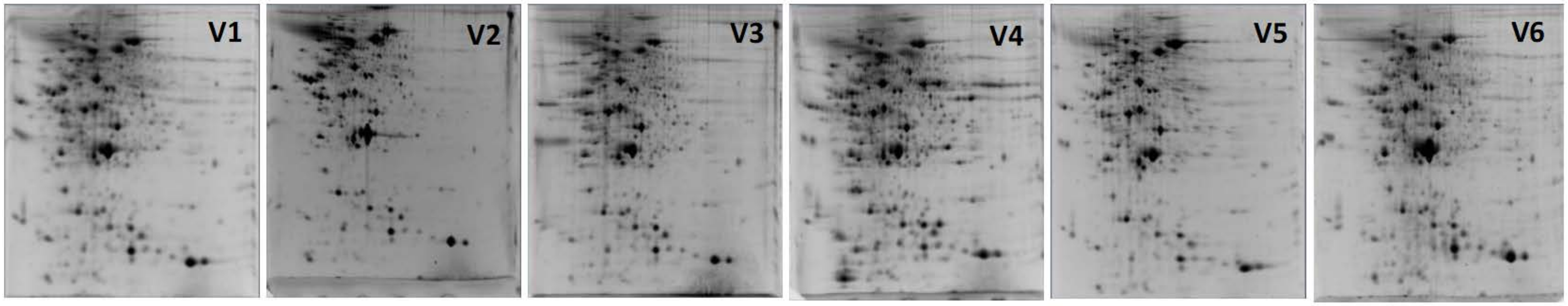
V1
V2
V3
V4
V5
V6

Supplementary Figure 2. Raw files from the BioRad Imager saved as JPEG files, uncropped. A yellow frame (V4) marks the figure used in Figure 2. A red frame is used for the picture used in Figure 3 in the paper (D2, C4, V1).

DORSAL FILES

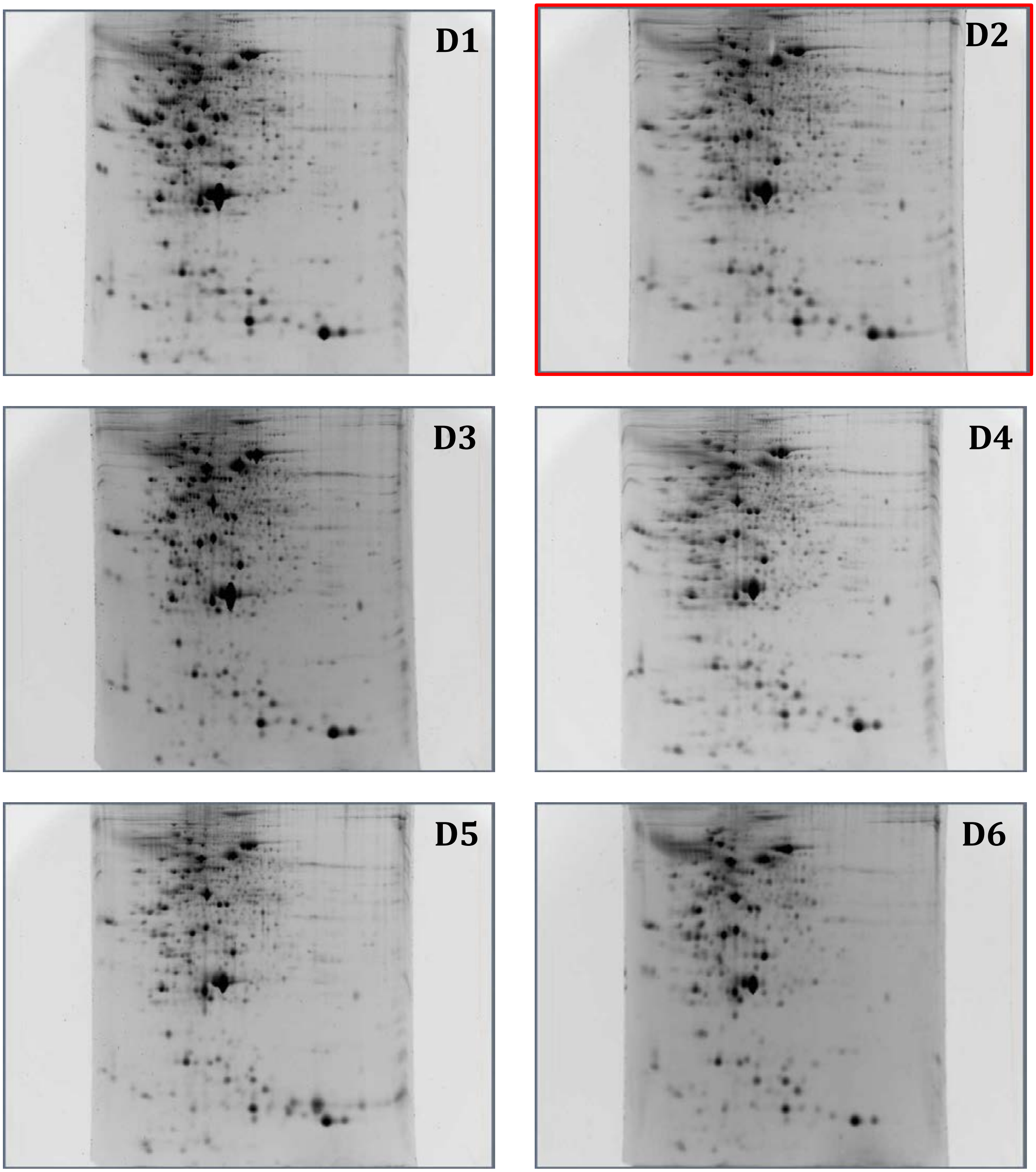

CAUDAL FILES

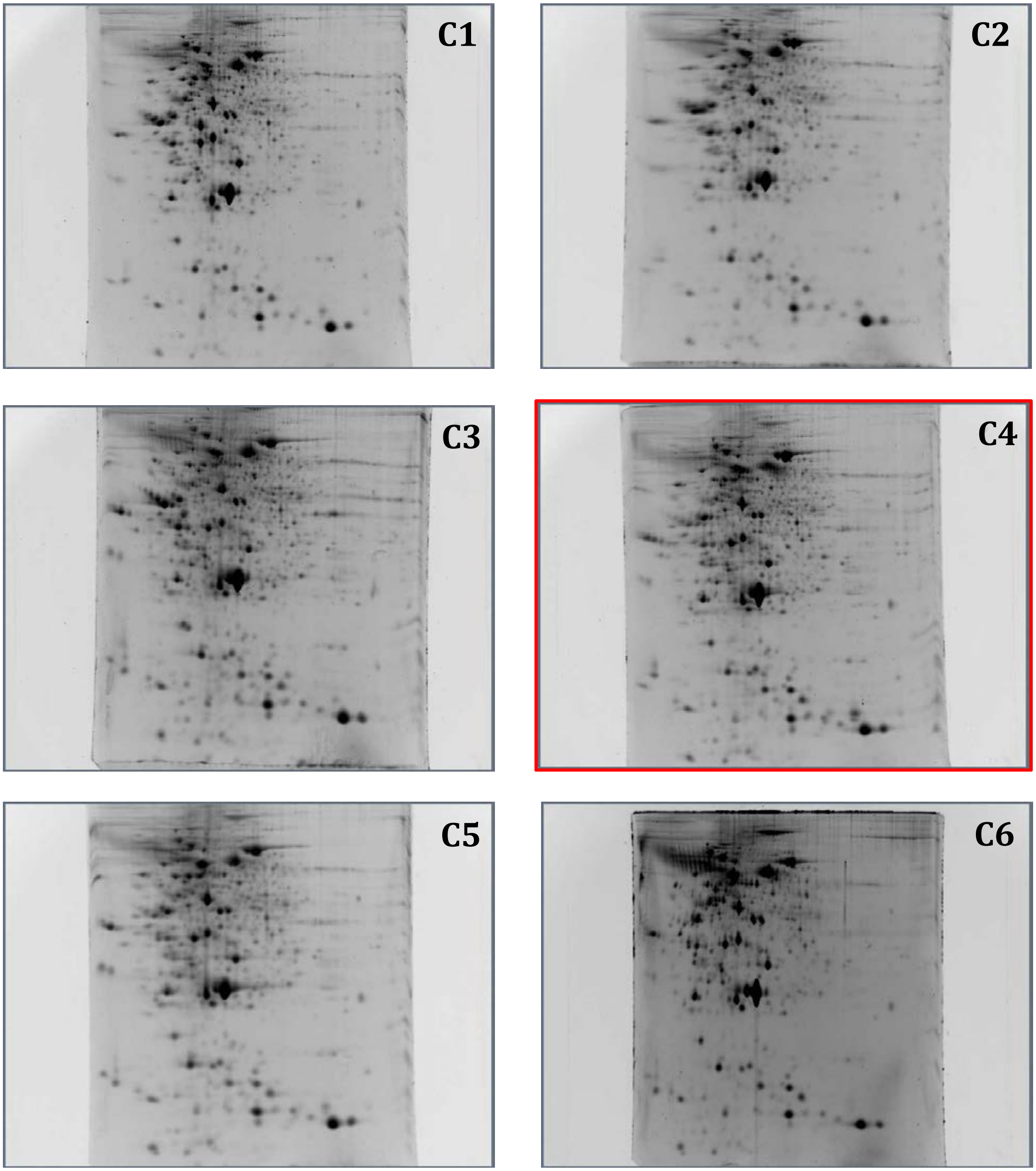

VENTRAL FILES

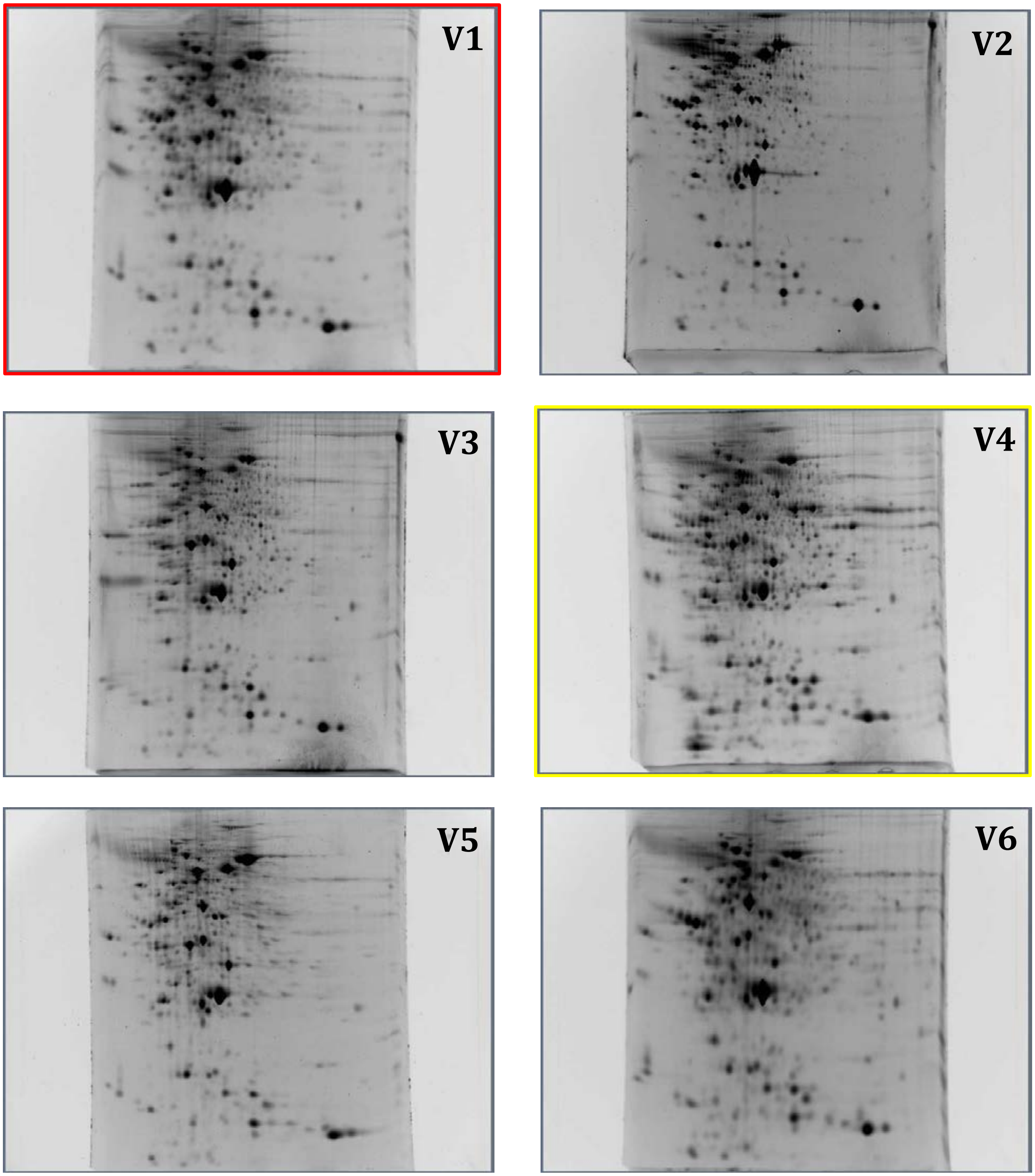

Supplement: Supplementary file 1 — Proteomic and structural differences in lumpfish skin among the dorsal, caudal and ventral regions [file 41598_2019_43396_MOESM1_ESM.pdf]
